# Fibronectin localization and fibrillization are affected by the presence of serum in culture media

**DOI:** 10.1038/srep09278

**Published:** 2015-03-23

**Authors:** Alessandro Siani, Rong R. Khaw, Oliver W. G. Manley, Annalisa Tirella, Francesco Cellesi, Roberto Donno, Nicola Tirelli

**Affiliations:** 1 Manchester Pharmacy School, University of Manchester, Oxford Road, Manchester, M13 9PT, United Kingdom; 2 School of Medicine, Institute of Inflammation and Repair, University of Manchester, Oxford Road, Manchester, M13 9PT, United Kingdom

## Abstract

*In vitro* models of fibrotic phenomena are often based on the fibroblast-myofibroblast transition as the contraction-triggering cellular event. There are, however, multiple sources of concern regarding the appropriateness of such models; a first and widely investigated issue is the often inappropriate nature of the interactions between mesenchymal cells and surrounding/underlying matrix/substrate. A second set of problems concerns the composition of the fluid phase, which includes both dispersed/dissolved paracrine messengers and matrix elements. In this study, we have focused on the effects that serum may generate. We have observed that A) serum causes high variability in the expression of typical markers of myofibroblast differentiation (ED-A fibronectin and α-Smooth Muscle Actin) upon treatment with TGF-β1; this is probably due to intrinsic variability of cytokine concentrations in different batches of serum. B) the fibrillization of endogenous fibronectin is partially hampered and its localization changed from ventral (on the substrate) to dorsal (upper surface); the latter morphology appears to be largely overlooked in literature, even though it may have a significant role in terms of mechanotransductive signaling. This quite dramatic change possibly occurs as a result of competition with serum proteins, although our data seem to rule out a direct role of serum fibronectin.

Myofibroblasts, a contractile phenotype of mesenchymal cells, are major players in the contraction phenomena that underpin both physiological wound healing and abnormal scarring, as well as a vast number of pathological fibrotic/foreign-body reactions^1^. The availability of appropriate *in vitro* models is a major goal in order to understand and control the mechanical and biochemical interactions of myofibroblasts with the environment surrounding them, and it is a fundamental step towards the development of new therapies. Mechanotransduction and matrix remodelling specifically play a key and inter-dependent role; for example, mechanical effects such as the stiffening of the extracellular matrix (ECM) can upregulate the fibroblast proteolytic activity^2^. In turn, matrix remodeling by enzymes such as Matrix MetalloProteinases (MMPs) can alter the balance of mechanical forces, with a knock-on effect on the organization of cell cytoskeleton^3^. However, the organization of mechanotransductive elements and MMPs in *in vitro* models has been relatively little studied.

The extracellular fibrillar network formed by fibronectin is one the most critical mechanotransductive components^4^, thanks to its physical continuity with actin fibres via transmembrane integrin association^5^. Indeed a distinctive splice variant of fibronectin, the ExtraDomain-A fibronectin (ED-A FN), is critical for acquisition of the myofibroblast phenotype^6^; ED-A FN is also commonly used as an early stage marker of this differentiation.

Literature also suggests some links between MMP expression and development of the myofibroblast phenotype, and most evidence seems to link MMP-2 (see Supplementary Information, Section 1, Table S1) and MMP-14 to contraction/fibrotic processes. MMP-2 expression appears to increase in hypertrophic and keloid scars^7^,^8^ and in fibrotic liver^9^,^10^, and seems to be more associated to the degradation of normal ECM (relatively richer in collagen IV) rather than that of a more fibrotic one (richer in collagen I)^11^. MMP-3 may also have a positive association with myofibroblasts: MMP-3 knockout decreases collagen gel contraction in primary mouse fibroblasts *in vitro*, impairs wound contraction in an *in vivo* mouse model^12^, and it has also been shown to induce Epithelial-Mesenchymal Transition (EMT)^13^. To our knowledge, limited data exist to support a link with MMP-1, although an all-in-all negative association may be possible: for example, Transforming Growth Factor β1 (TGF-β1) treatment decreases MMP-1 expression in keloid fibroblasts, where the reverse occurs upon TGF-β1 inhibition^14^. Increased MMP-1 and reduced collagen levels are also associated to an improved wound healing caused by basic fibroblast growth factor (bFGF)^15^. Finally, MMP-1 levels increase when Smad interacting protein 1 (SIP1) is overexpressed, which is in turn repressed by TGF-β1 in both normal and pathological scar fibroblasts^16^.

However, the variability of the *in vitro* experimental data is a major issue and frustrates efforts to reach solid conclusions: for example, most reports show MMP-2 upregulation by TGF-β1, but some claim downregulation^17^,^18^. Similar considerations can be made also for the quantification of classical markers of the TGF-β1-induced myofibroblast transition; for example, in a seminal paper on myofibroblast differentiation Gabbiani reported that the same TGF-β1 concentration induced higher α-Smooth Muscle Actin (α-SMA) levels with decreasing serum concentration (FBS or WBS)^19^. Therefore, it seems reasonable to question whether *in vitro* models exist that allow a reproducible myofibroblast generation and a recapitulation of their *in vivo* behavior. Firstly, most models lack a biological or at least biomimetic ECM; this is a key point, since *in vivo* a general MMP inhibition causes delayed myofibroblast differentiation, while this effect is absent on TGF-β1-treated fibroblast cultures^20^. Secondly, most data are gathered from cells cultured on plastics and exposed to TGF-β1, but there is no general consensus about the necessity of the presence of serum in the medium, nor about its concentration. Clearly, serum-free conditions provide an extremely artificial environment that can hardly mimic the (signalling) complexity of a biological fluid. On the other hand, the high variability of components present in serum may significantly influence myofibroblast differentiation and behavior.

Here, we have investigated the effect of a serum-containing medium (10% FBS) on the expression of markers of myofibroblast differentiation and on the (co)localization of structural elements (actin, fibronectin) and of MMPs.

## Results

### Influence of serum on myofibroblast markers

**Figure 1:**
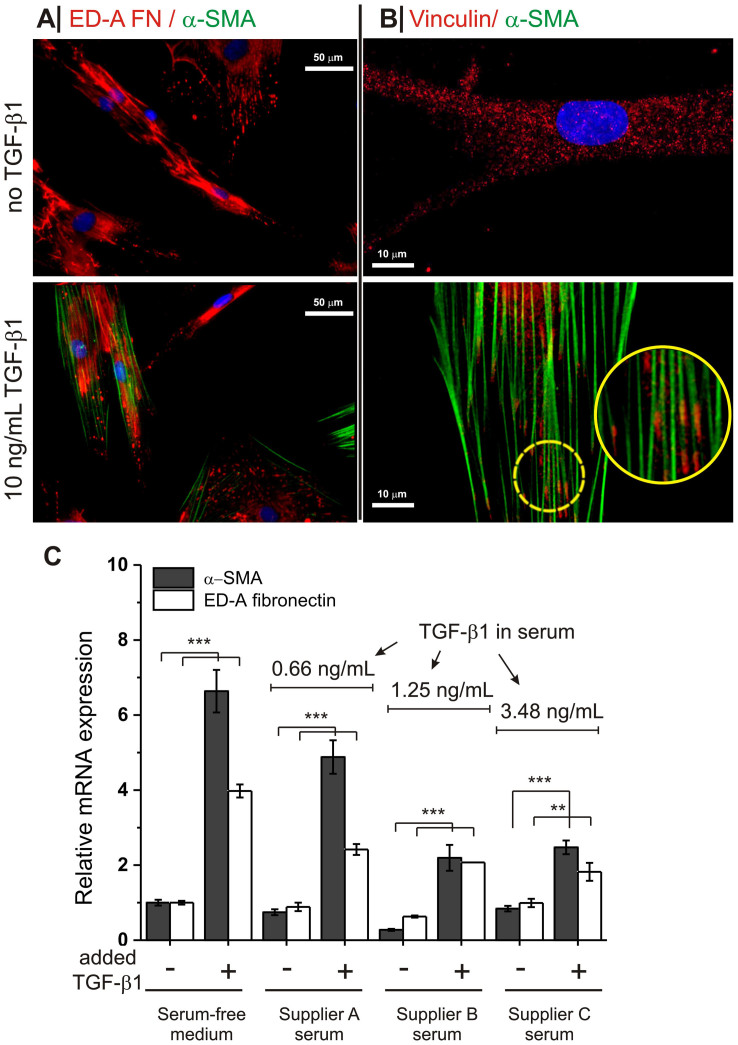
Expression of (proto)myofibroblastic markers EDA-FN and α-SMA in response to TGF-β1. (*A*): HDFs showed significant amounts of ED-A FN already before TGF-β1 treatment, which is a sign of a protomyofibroblastic phenotype. TGF-β1 clearly induced the development of α-SMA in these cells. (*B*): Vinculin staining showed that TGF-β1 treatment not only induced the formation of α-SMA-containing stress fibres (green) in HDFs, but also of focal adhesions at their termini (red, magnified in the circles). (*C*): *Top*: TGF- β1 concentration in media containing FBS from different sources, which are anonimized; please note that supplier A was used for the experiments in panels A and B and for all experiments reported in the rest of the paper. *Bottom*: The exposure of HDFs to a 10 ng/mL TGF-β1 dose caused different upregulation of myofibroblast markers depending on the actual TGF-β1 content in the culture medium (measured via ELISA). *n* = 3 for all experiments (two stars: p-value < 0.01, three stars: p-value < 0.001, obtained through a two-tailed, unpaired T-test).

Due to their consistent TGF-β1-induced overexpression of both the protomyofibroblast marker ED-A FN and the myofibroblast marker α-SMA (Figure 1A), human dermal fibroblasts (HDF) were selected as the cellular model for this study.

Please see Supplementary Information, section 1, Figure S1, for a comparison of HDFs with two other common fibroblastic cell types (L929 and 3T3 murine cell lines). However, the use of HDFs carries two important *caveats*: A) HDFs are already in a protomyofibroblastic (ED-A FN positive) state before TGF-β1 treatment, possibly due to the stiffness of the plastic substrate. B) the TGF-β1 –stimulated emergence of myofibroblast markers in serum-containing media was very variable, with overexpression levels ranging from 2- to 8-fold. We related the variations in the marker expression to differences in the supplier and probably in the batch of serum. Indeed, ELISA analysis showed that sera from different suppliers contained a highly variable amount of TGF-β1 (up to 1 ng/mL), which appeared to have an inverse correlation with the marker expression (Figure 1C). Possibly, the variable TGF-β1 level in commercial sera is one of the sources of the variability observed in Table S1.

It is worth mentioning that the most reproducible upregulation of both markers was obtained using a serum-containing medium only to favour cell attachment, and then performing the TGF-β1 treatment under serum-free conditions.

### Influence of serum on the morphology of structural elements

**Figure 2:**
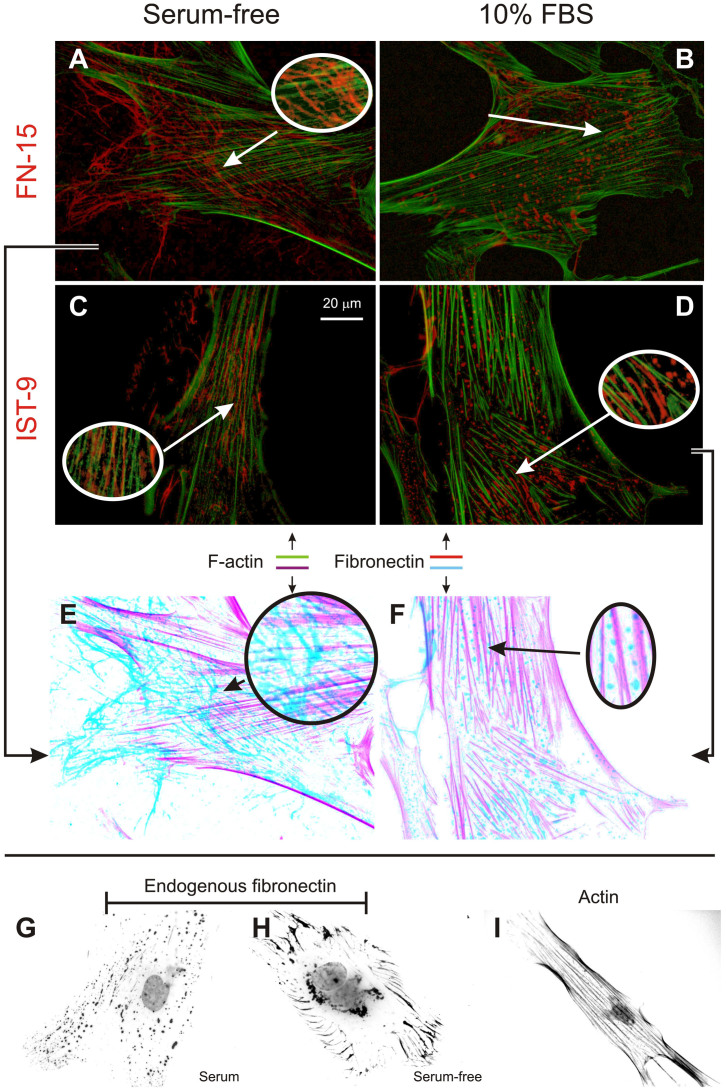
FN staining on HDFs. “General” FN was stained via FN-15 antibody (*A* and *B*) and ED-A FN via IST-9 (*C* and *D*, 10 ng/mL TGF-β1), and both exhibited a fibrillar organization without serum (*A* and *C*), and a predominantly non-fibrillar one under 10% FBS. Oval insets show magnified views of the interconnection between fibronectin (red) and actin (green) in the absence of serum, while they appear mutually exclusive in its presence. Panels *E* and *F* represent the images in *A* and *D* in inverted colours (cyan for FN, magenta for actin) for a clearer appreciation of spatial relation of fibronectin with actin. Panels *G* to *I* summarize the different morphology of endogenous FN with serum (spotted, *G*) or without serum (fibrillar, *H*) and actin (fibrillar but with a different pattern); images *G* to *I* were obtained by first inverting the colours of DAPI+actin/FN-stained cells and then converting them to grayscale.

**Figure 3:**
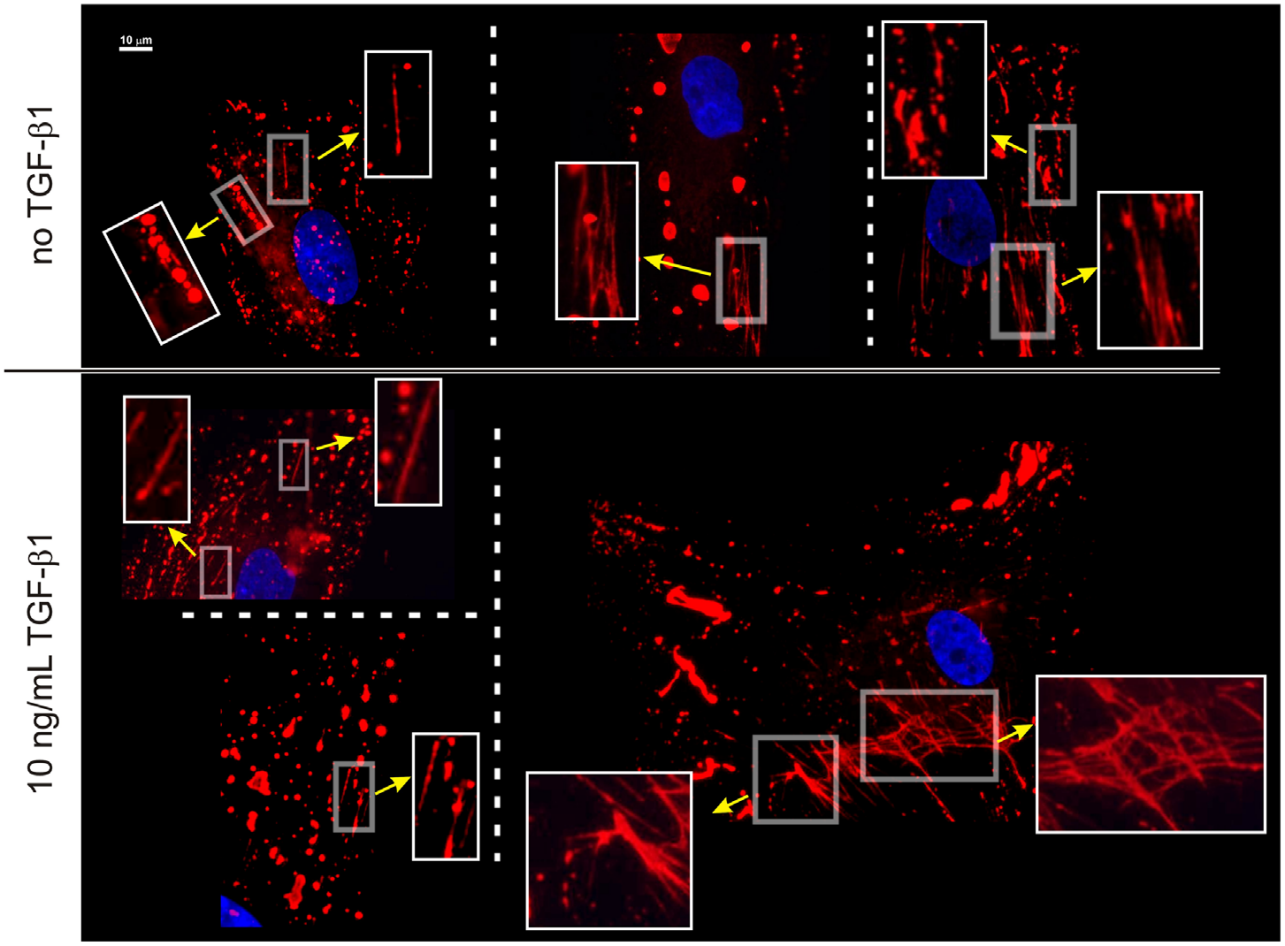
Morphology of endogenous ED-A FN for HDFs cultured in a serum-containing medium. The shape is predominantly irregular and featureless; however, independently on the treatment with TGF-β1, areas of partial fibrillization are often recognizable in up to 30–40% of the cells in any given population. The insets show magnified views of these areas In particular the images on the left could be consistent with a pre-fibrillar organization from aligned “spots”; however, we are inclined not to follow this hypothesis, due to the dorsal/ventral differential localization of respectively non-fibrillar and fibrillar FN (see Figure 4).

Both F-actin in untreated fibroblasts, and α-SMA in TGF-β1-treated ones showed no apparent morphological difference in the presence or absence of serum (see Figure 2 for an example of F-actin in both conditions). On the other hand, serum strongly affected the spatial organization of fibronectin (FN), both in a “general” form (hereafter referred to simply as FN) and in its ED-A variant, as respectively stained with the FN-15 and IST-9 monoclonal antibodies (Figure 2). Both with and without TGF-β1 treatment, FN and ED-A FN predominantly adopted a fibrillar organization in the absence of serum, and a non-fibrillar, spot-like morphology in 10% FBS. Note that this does not necessarily imply the complete absence of fibrils in serum-containing media (Figure 3), nor the absence of ‘spots’ in a serum-free environment: HDFs always showed both FN morphologies, although in different relative amounts depending on the culture conditions. Elongated structures were often seen, which may relate to a transition from “spots” to fibrils; these structures generally appear co-aligned but not overlapping with along actin fibres (see e.g. insert in Figure 2D). It is also noteworthy that FN topology was indistinguishable after 2, 3 and 5 days of culture in the absence of serum (see Supplementary Information, Figure S2), therefore indicating a 2-day starvation is sufficient to induce the morphological difference.

**Figure 4:**
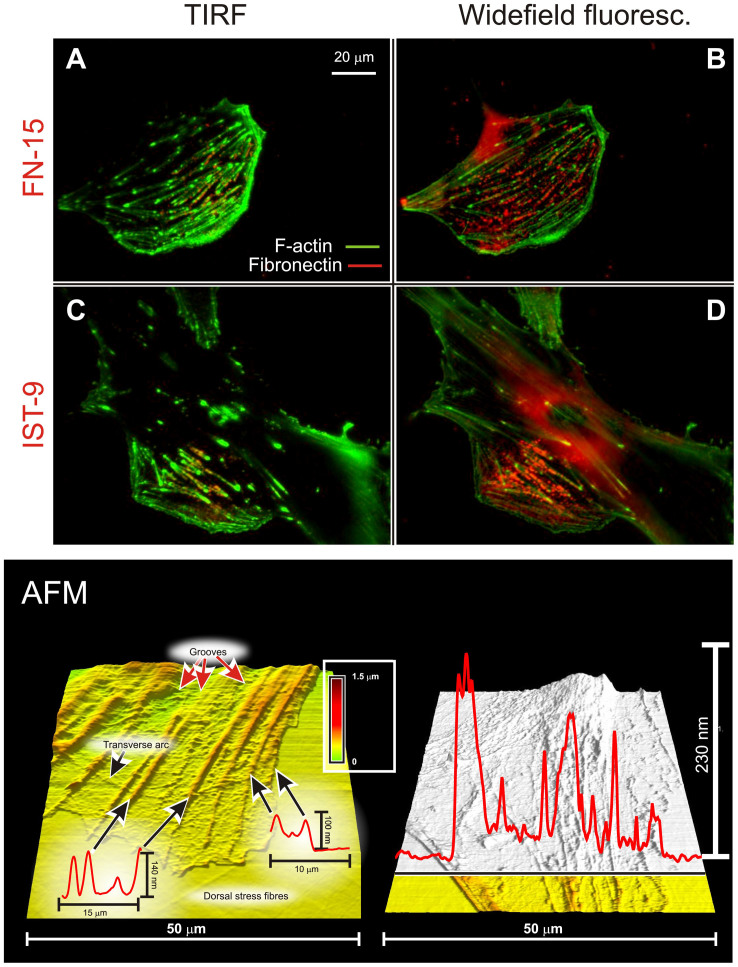
Localization of fibronectin in HDFs cultured in 10% FBS. *Top*: In 10% FBS both “total” FN (imaged with the FN-15 antibody, *A* and *B*) and ED-A FN (imaged with the IST-9 antibody, *C* and *D*) showed a strong epifluorescence emission, but a weak TIRF one. F-actin (green) showed a stronger TIRF emission due to the localization of many stress fibres on the ventral surface of HDFs. Please note that FN appears to be present in a perinuclear region in the two epifluorescence pictures; although frequent, this localization was not observed in all samples. *Bottom*: contact mode AFM pictures of fixed HDF in PBS. The cell surfaces present ridges with heights at most of a couple of hundreds of nm, which correspond to the dorsal actin fibres (smaller for transverse arcs); a similar picture is presented in Supplementary Information, Figure S3 (see Section 2 in Supplementary Information, Additional Materials and Methods for experimental details). The picture in the right hand part shows the full contour of a peripheral part of an HDF body, as highlighted by the black line); in areas where actin fibres are not present, the thickness decreases to less than 50 nm, which explains the bleeding of the dorsal fluorescence on the ventral plane in Figure 5.

**Figure 5:**
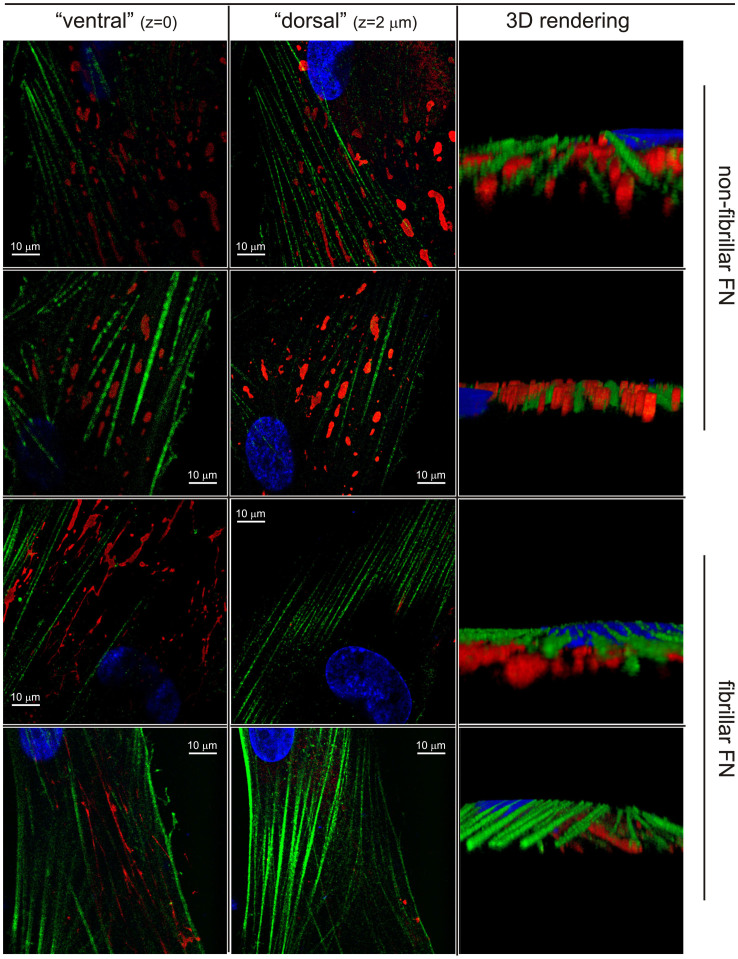
Confocal microscopy of ED-A FN (red) imaged with the IST-9 antibody. The non-fibrillar form of FN appears to be present dorsally and localized between neighboring actin fibres (green); within the resolution of the technique, there is no apparent difference in vertical position between actin and FN. It should be noted that the low intensity ventral red fluorescence is actually due to dorsally localized components that cannot be completely separated due to a cell thickness (<100 nm, see AFM pictures in Figure 4, bottom) of the same order of magnitude of a confocal voxel. On the contrary, the fibrillar form recorded in serum-free medium was clearly positioned ventrally, in between cell body and substrate.

In terms of localization, in serum the non-fibrillar form of FN appeared to be predominantly present on the upper (dorsal) surface. For example, it was more visible in widefield than in TIRF images (Figure 4, top). Since actin appears to be negatively colocalized with FN/ED-A FN ( = mutual exclusion; see e.g. Figure 2F or Figure 4), we assume the FN “spots” to be located in the grooves between stress fibres on the upper cell surface (Figure 4, bottom), which would explain their alignment directed along that of the actin fibres (see e.g. Figure 2F). The non-fibrillar FN was localized dorsally also under serum-free conditions, as confirmed also by confocal analysis, whereas the more common fibrillar form localized ventrally at the cell/substrate interface (Figure 5).

In the absence of cells, FN morphology depends on the characteristics of the materials it can bind to 21,22,23; when in contact with cells, the major determinant of FN fibrillization is its association to integrins^24^. This applies to all forms of cellular FN, including ED-A FN: for example, a largely non-fibrillar ED-A FN was recorded in lymphatic vessels of mice lacking α9 integrin^25^. It could be hypothesized that the reduced FN fibrillization observed in serum may be caused by integrins adhering to the substrate through interactions with competing molecules from the medium: for example, 20 nM FN in the medium is sufficient to provide a fibrillar mesh for FN-null fibroblasts^26^. We have therefore supplemented the serum-free medium with up to 57 nM bovine FN, but this caused no apparent alteration to the fibrillar staining pattern of FN15 nor the presence of non-fibrillar human FN; this would suggest that serum components other than FN may lead the adhesion the cause of the lack of fibrillization (see Supplementary Information, Figure S5).

The ED-A FN (IST-9 positive) and “total” FN (FN-15 positive) showed an indistinguishable morphology and spatial distribution; since they are both murine anti-human monoclonal antibodies and therefore cannot be used in combination, we have only followed the former for the rest of the study.

### Spatial relation of non-fibrillar FN to other structures (serum-containing medium)

We have first focused on MMP-2. This protease has a certain degree of structural similarity to FN, with three fibronectin-like domains allowing its binding to collagen/gelatin^27^,^28^,^29^. MMP-2/FN colocalization has been occasionally reported, e.g. in infected astrocytes^30^ and may arise from binding to/competition for a common substrate; for example, when HDFs are cultured on collagen IV, the colocalization of the substrate with fibrillar FN^31^,^32^ is possibly coupled to that with MMP-2, which eventually degrades FN^31^. This ternary collagen IV/MMP-2/FN interplay may be influenced by the FN morphology: MMP-2 is decreasingly activated with increasing binding of fibronectin by integrins, which should cause FN increased fibrillization^33^.

The question is therefore whether non-fibrillar FN may be associated to gelatinolytic sites, i.e. MMP-2 or MMP-9. We have employed DQ™ (dye-quenched) gelatin, which allows for a fluorimetric detection of gelatinase activity. Incidentally, HDFs have significant gelatinolytic activity in both protomyofibroblastic and myofibroblastic state, as demonstrated also via zymography (see Supplementary Information, Figure S4); this may correct earlier reports that ascribed the gelatinase activity of fibrotic samples only to macrophages^34^.

**Figure 6:**
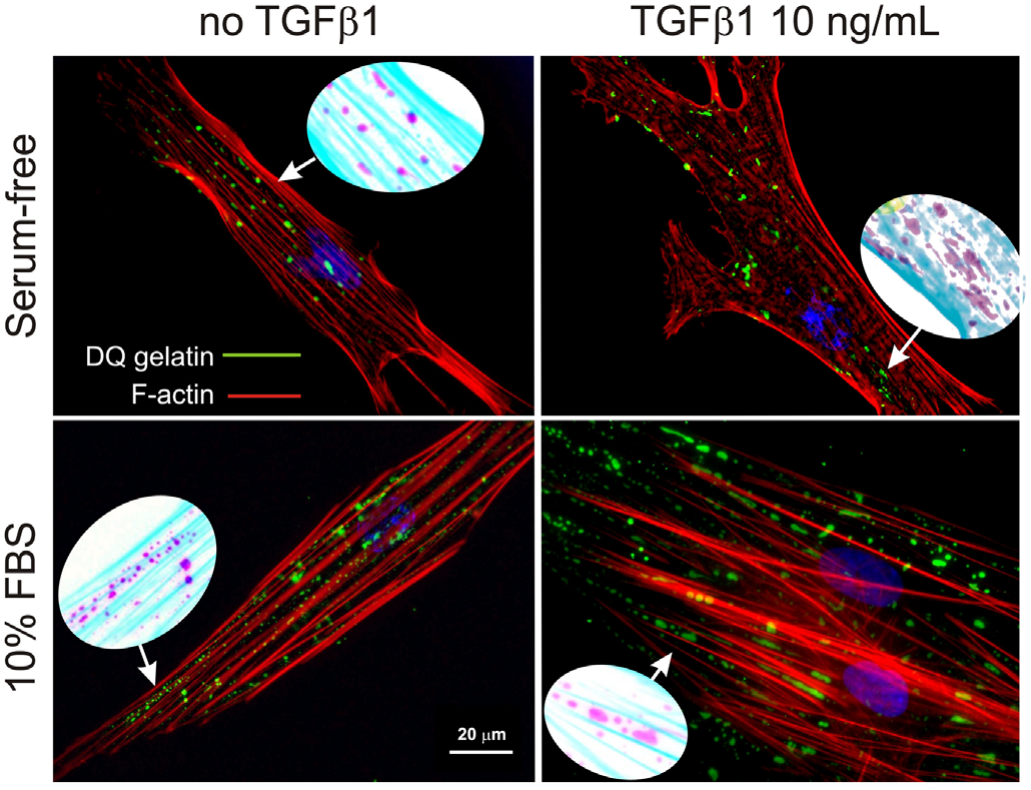
Localization of gelatinolytic sites (green fluorescence) and actin (red fluorescence) for HDFs cultured under serum-free conditions (*top*) or in 10% FBS (*bottom*) and with (*right*) or without (*left*) 10 ng/mL TGF-β1. DQ™ gelatin fluorescein groups are quenched in the macromolecule prior to degradation due to its high degree functionalization, whereas they fluoresce upon enzymatic cleavage with an intensity proportional to the extent of degradation. The oval insets show magnified portions of the pictures in inverted colours for an easier appreciation of the localization of DQ™ gelatin fluorescence predominantly between actin fibres.

Irrespective of the presence of serum or TGF-β1, DQ™ gelatin showed a spotted, actin-avoiding fluorescence, with a pattern very reminiscent of that of non-fibrillar FN (Figure 6).

**Figure 7:**
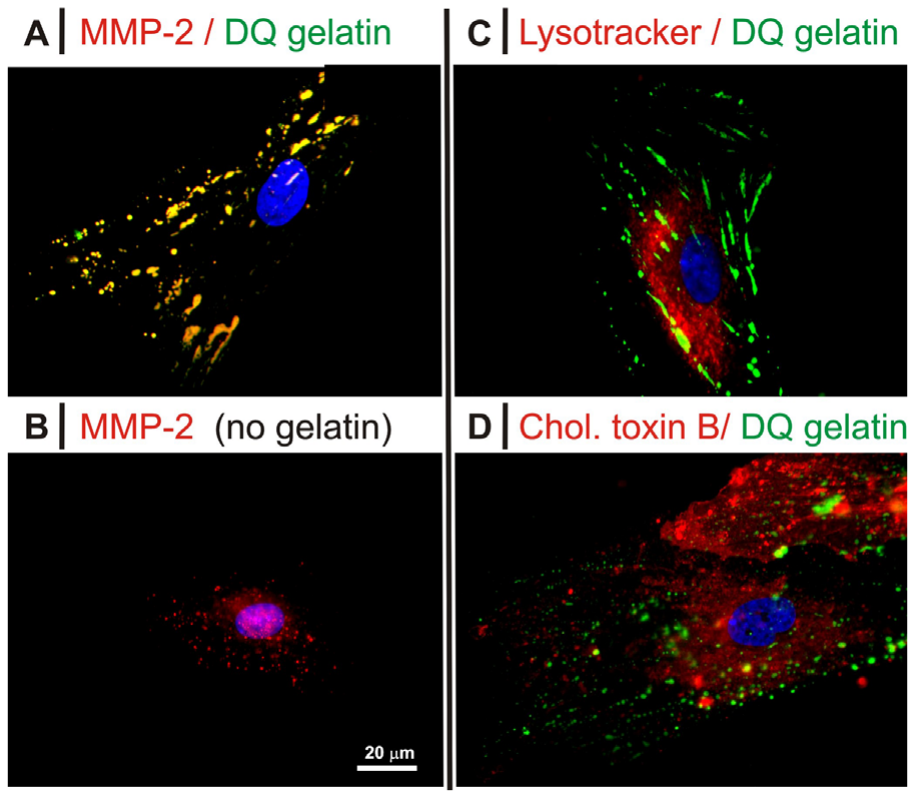
Fluorescence microscopy images of HDFs exposed to DQ™ gelatin in 10% FBS without TGF-β1. In all images green fluorescence corresponds to gelatin degradation. Red fluorescence corresponds to MMP-2 (rabbit polyclonal antibody and anti-rabbit Chromeo™) in (*A*) and (*B*), to endolysosomal compartments (Lysotracker) in (*C*), to lipid rafts (Cholera Toxin B) in (*D*). Please note that 10 ng/mL TGF-β1 provided indistinguishable results, see Supplementary Information, Figure S6.

Indeed, gelatinolytic sites showed an almost complete colocalization with MMP-2 both before (Figure 7A, see also Supplementary Information, Figure S7) and after TGF-β1 treatment (see Supplementary Information, Figure S6A and Figure S8); in the absence of gelatin, MMP-2 presented only a diffuse cytoplasmic staining pattern (Figure 7B and Supplementary Information, Figure S6B), thus the protease apparently migrated to the cell surface in a substrate-dependent fashion. It is worth pointing out that DQ™ gelatin was incubated overnight, but despite the rather long incubation we are confident that MMP-2 and degraded DQ™ gelatin did not undergo significant internalization: A) DQ™ gelatin did not colocalize with late endosomes (Figure 7C and Supplementary Information, Figure S6C); B) MMP-2 endocytosis is possibly associated to lipid rafts (the low density lipoprotein receptor-related protein-1 (LRP-1) internalizes MMP-2^35^,^36^,^37^ and is stabilized via cholesterol enrichment^38^), but no significant colocalization between them and DQ™ gelatin/MMP-2 was recorded (Figure 7D and Supplementary Information, Figure S6D).

**Figure 8:**
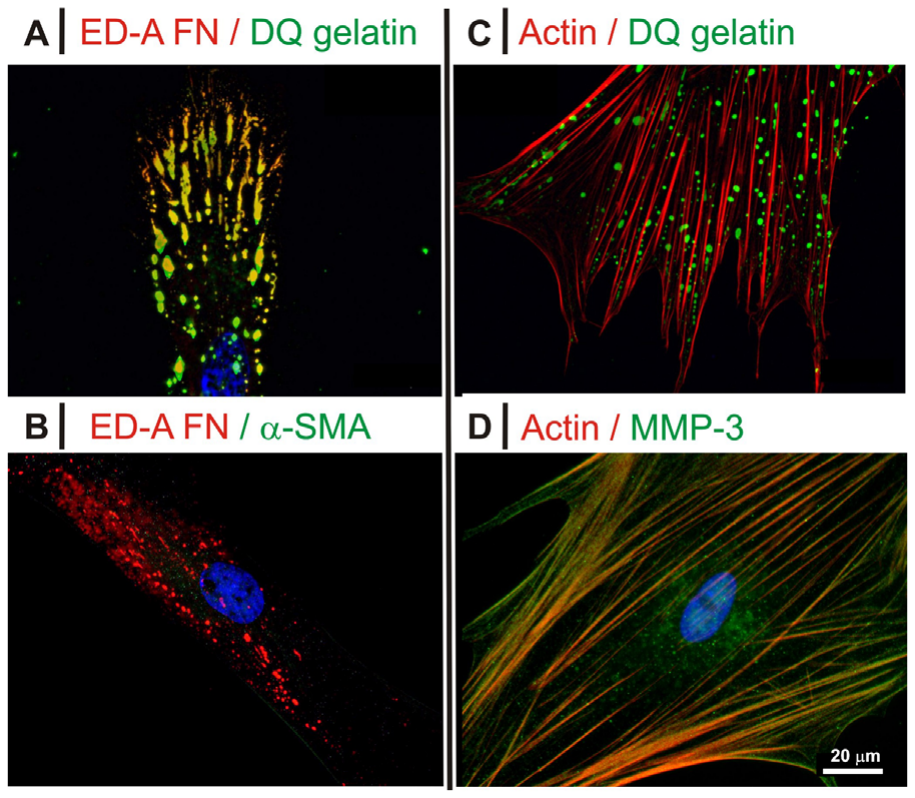
Fluorescence images for HDFs cultured in 10% FBS without TGF-β1. (*A*): ED-A FN (red) and degraded gelatin (green) showed an almost perfect colocalization; please note, although in this part of the study we have focused on ED-A FN, FN and ED-A FN share the same localization pattern (Figure 2). (*B*): α-SMA was substantially absent without TGF-β1, but in myofibroblasts it mapped the localization of F-actin and thus showed a virtually complete exclusion with ED-A FN. (*C*): Evidence of mutual exclusion between actin and DQ™ gelatin, as shown also in Figure 5. (*D*): MMP-3 (green) showed a predominantly fibrillar distribution that colocalized with that of actin (red), although some protease also accumulated in the perinuclear area. The separate green and red channels for all pictures are presented in Supplementary Information, Figure S7, right. Please note that 10 ng/mL TGF-β1 provided indistinguishable results, see Supplementary Information, Figure S8.

Therefore it seems safe to assume that it is still on the cell surface that the gelatinolytic sites positively colocalized with non-fibrillar FN (ED-A FN in Figure 8A) whereas actin/α-SMA negatively colocalized with both (Figure 8B and C, see also Supplementary Information, Figure S7 and S8). Please note that for ease of performance and higher resolution, the colocalization was analyzed on epifluorescence rather than confocal images, due to the thinness of the cell body (<200 nm, similar to the voxel size).

**Figure 9:**
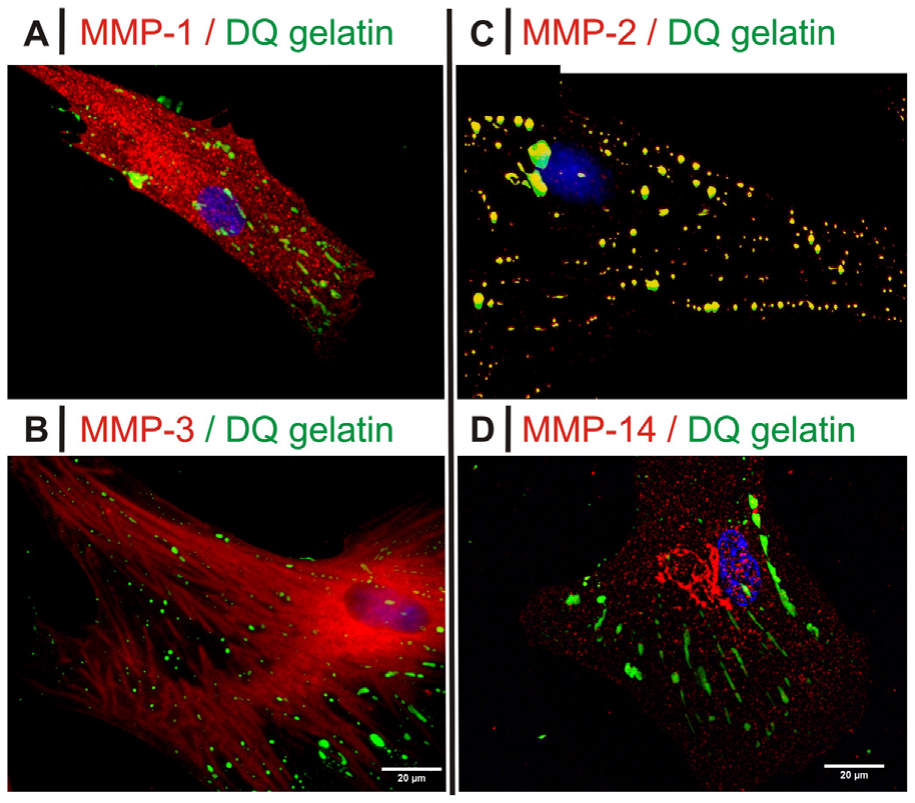
Fluorescence images for HDFs exposed to DQ™ gelatin in 10% FBS without TGF-β1. In all images the green fluorescence corresponds to gelatin degradation. The red fluorescence corresponds to MMP-1 (*A*), MMP-3 (*B*), MMP-2 (*C*) and MMP-14 (*D*); for the latter, the fluorescence pattern would suggest an intracellular/perinuclear localization (shape reminiscent of the Golgi). The separate green and red channels for all pictures are presented in Supplementary Information, Figure S7, left. Please note that 10 ng/mL TGF-β1 provided indistinguishable results, see Supplementary Information, Figure S8.

Extending the study to other MMPs, we observed that MMP-3 mapped the F-actin localization (Figure 8D; see also Supplementary Information, Figure S7 and S8) and avoided MMP-2/DQ™ gelatin/ED-A FN areas (Figure 9B and C). MMP-1 and MMP-14 showed no preferential spatial association with either actin or FN (Figure 9A and D; see also Supplementary Information, Figure S7 and S8).

A quantitative analysis confirmed the serum-induced colocalization of DQ™ gelatin and ED-A FN, and its mutual exclusion with MMP-3 and actin (see Supplementary Information, Table S2 and Figure S9). It is noteworthy that replacing 10% FBS with serum-free medium the colocalization of ED-A FN and actin changed from mutually exclusive to random (see also Figure 2), whereas DQ™ Gelatin and actin kept their mutually exclusive distribution.

## Discussion

A comparison with recent literature makes it possible to draft some tentative links:

### A) Positive colocalization between non-fibrillar FN and MMP-2 and mutual exclusion with actin

First, recent evidence supports this finding; for example, Jacob et al. showed positive and negative colocalization respectively for gelatin/fibronectin and for MMP-2/actin in MDA-MB-231 cells cultured on a gelatin-coated substrate^39^. Further, MDA-MB-231 invadopodia showed gelatinolytic sites with a pattern analogous to our observations, which were surrounded by but did not overlap with F-actin^40^.

Second, in a serum-containing medium the positive MMP-2/FN colocalization may be the result of a functional link. ED-A FN was predominantly non-fibrillar irrespective of the presence of gelatin. When added, the latter adopted the ED-A FN morphology and MMP-2 follows suit, possibly suggesting that gelatin/FN binding precedes or even causes the MMP-2 surface migration; this may explain the significantly reduced MMP-2 expression in ED-A FN knockout mice^41^. In the absence of serum, the fibrillar FN no longer showed a preferential colocalization with gelatin and therefore differences in FN-based signalling are possible.

Third, also the actin/MMP-2 negative colocalization may stem from a functional link, albeit unknown. Some literature reports point in this direction; for example, MMP-2 activation increased upon treatment with the actin-disrupting cytochalasin D in human trabecular meshwork cells^42^, in human palmar fascia fibroblasts and in human foetal lung fibroblasts^43^, whereas the disruption of microtubules (via nocodazole) did not have an effect on the protease. In a reciprocal fashion Jin et al. showed that Akt-mediated activation of MMP-2 in chick wing buds mesenchymal cells resulted in actin cytoskeleton reorganization^44^.

### B) Positive colocalization between actin and MMP-3

To the best of our knowledge, a topological association between MMP-3 and cytoskeletal elements has never been reported, but there are evidences of a close functional relationship: for example, MMP-3-deficient fibroblasts showed an impaired contraction *in vitro*^45^, whereas MMP-3 knockout mice presented an impaired early wound contraction due to inadequate actin organisation in fibroblasts^46^. Additionally, it has been shown that in the contraction of collagen gels by osteoblasts MMP-3 (and MMP-1) and α2 integrins follow a similar path of expression^47^.

### C) No difference following TGF-β1 treatment

It is noteworthy that two HDF phenotypes were used, i.e. protomyofibroblastic (no TGF-β1, but yet ED-A FN expression) and myofibroblastic (10 ng/mL TGF-β1, with expression of both ED-A FN and α-SMA); however, this did not affect the distribution and morphology of any of these structural or functional elements (compare Figure S7 and S8 in Supplementary Information).

## Conclusion

A serum-free (myo)fibroblast model is definitely artificial, but how appropriate and reproducible is one *with* serum? Our results point out that it may be equally artificial and possibly less reproducible. In short, the two main *caveats* are a) the presence of variable amounts of TGF-β1 (and possibly other cytokines), which leads to variable levels of overexpression of myofibroblast markers, and b) the reduced level of fibrillization of endogenous FN, possibly because of the presence of other serum-borne factors that compete for integrin binding, which may also have downstream effects in terms of cellular signaling.

Incidentally, proteolytic sites and structural elements appeared to have identical topological links in protomyofibroblasts and in myofibroblasts; this would indicate that, despite their different contractility, the two cell types may have a similar mechano-transducive machinery controlling their interaction with the extracellular matrix.

## Methods

### Chemical reagents and general cell culture

Primary human dermal fibroblasts (HDFs) (C-013-5C) were purchased from Cascade Biologics/Invitrogen (Paisley, UK) and used within passage 3–6 from reception. HDF were cultured using high glucose Dulbecco's Modified Eagle Medium (DMEM, D6546, Sigma-Aldrich) supplemented with 10% v/v foetal bovine serum (FBS, Invitrogen), 1% v/v penicillin/streptomycin solution (Sigma-Aldrich), 1% v/v L-glutamine (Invitrogen) and incubated under sterile conditions at 37°C/5% CO_2_. For all experiments, cells were seeded in the appropriate culture vessel and allowed to attach for 24 hours in presence of 10% serum. Subsequently, they were washed 3 times with warm serum-free medium (SFM), and treated for 48 hours with TGF-β1 (human recombinant, Abcam, UK) or bovine fibronectin (F1141, Sigma) in SFM or 10% serum, according to the desired experimental conditions. Please note that the use of human TGF-β1 in murine fibroblast cell lines is a common practice^48^,^49^,^50^,^51^, due also to its close homology to the murine growth factor (identical 390 aa length and 89.7% identity). For fluorimetric quantification (e.g. DAPI or DQ™ gelatin) cells were grown on black 96 well plates (Greiner Bio-One), and fluorescence was measured with a Safire microplate reader (Tecan, Männedorf, CH). Excitation/emission maxima for DAPI and DQ™ gelatin were 358/461 nm and 485/520 nm, respectively.

### Experimental descriptions for RNA analysis and qRT/PCR, Atomic Force Microscopy (AFM), gel zymography and quantitative colocalization analysis

See Supplementary Information, section 2 (Comparison of the effects of TGFβ1 exposure in murine cell lines and in HDFs), 3 (AFM on cells), 4 (Gelatin degradation) and 5 (Colocalization analysis).

### Enzyme-linked immunosorbent assay (ELISA)

The TGF-β1 content of sera sourced from different manufacturers was assayed using the Novex® TGF-β1 Multispecies ELISA Kit (Life Technologies, Paisley, UK) according to the manufacturer's instructions. The enzyme-mediated transformation of the chromogen in a coloured product was assayed measuring the 450 nm absorbance of each well using a Synergy 2 Biotek microplate reader. A calibration curve was built via serial dilutions of a supplied TGF-β1 standard. All absorbance measurements fell within the linear portion of the standard curve, thus ensuring the accuracy of the extrapolation.

### Immunocytochemistry and fluorescence microscopy

Cells were seeded at a density of 5 × 10^3^ cells/mL in slide flasks (Nunc-Fisher Scientific, Loughborough, UK) and were subjected to overnight incubation with 100 μg/mL of highly quenched fluorescein-labeled gelatin (DQ™ Gelatin D-12054, Molecular Probes®, Eugene, Oregon). The cells were then washed with PBS and fixed with 4% v/v paraformaldehyde/PBS for 30 minutes. For immunocytochemistry and phalloidin staining, permeabilization was performed using 0.25% v/v Triton X-100/PBS (Sigma-Aldrich) for 30 minutes followed by blocking using 5% v/v goat serum/PBS (Sigma-Aldrich) for 1 hour. Incubation of the following primary antibodies (all purchased from Abcam, Cambridge, UK) was performed at room temperature for 1 hour in 5% v/v goat serum/PBS: MMP-1 (rabbit polyclonal, 1:200), MMP-2 (rabbit polyclonal, 1:200), MMP-3 (rabbit polyclonal, 1:200), MMP-14 (rabbit polyclonal, 1:200), vinculin (SPM227 mouse monoclonal, 1:200), α-SMA (rabbit polyclonal, 1:200), ED-A FN (IST-9 mouse monoclonal, 1:200), total cellular fibronectin (FN-15, mouse monoclonal, 1:200) and bovine fibronectin (AB2047, rabbit polyclonal, 1:200). The cells were rinsed in PBS before incubation with the corresponding secondary antibodies: anti-rabbit Chromeo™ 488-conjugated secondary antibody (1:1000, goat polyclonal), anti-mouse Chromeo™ 546-conjugated secondary antibody (1:1000, goat polyclonal), anti-mouse Chromeo™ 488-conjugated secondary antibody (1:1000, goat polyclonal) in PBS for 1 hour. F-actin fibres were stained using 1:40 Alexa Fluor® 594-/488-conjugated phalloidin (Invitrogen) in 1% w/v bovine serum albumin/PBS for 1 hour. Late endosome/lysosome staining was performed by using 1 μM LysoTracker® Red DND-99 (Molecular Probes) for 1 hour, according to the manufacturer's instructions. Lipid rafts were stained with 25 μg/mL Alexa Fluor® 594-conjugated Cholera Toxin-B (CT-B, Invitrogen) for 1 hour in 5% w/v bovine serum albumin (BSA, Sigma-Aldrich). Cells were then mounted and counterstained using VECTASHIELD® mounting medium with 4′,6-diamidino-2-phenyl-indole (DAPI) (Vector Laboratories, Peterborough, UK).

Widefield images with 16-bit resolution and pixel size of 0.44 μm were obtained using a Olympus BX51 upright widefield microscope with a 60x/0.65–1.25NA Plan Fln oil immersion objective (Tokyo, Japan) and captured using a Coolsnap ES camera (Photometrics, Tucson, Arizona, USA) through MetaVue*™* Research Imaging Software (Molecular Devices, Sunnyvale, California). Specific band pass filter sets for DAPI (excitation: BP350/50 nm; emission: BP460/50 nm), fluorescein isothiocyanate (FITC; excitation: BP480/40 nm; emission: BP535/50 nm), and Texas Red (excitation: BP560/55 nm; emission: BP645/75 nm) were used to prevent bleed-through from one channel to the next. For Total Internal Reflection Fluorescence (TIRF) experiments, images were collected on a TE2000 microscope (Nikon), equipped with a perfect focus system to eliminate focus drift, using a 100x/1.49 Apo TIRF objective. The 488 nm laser line was manually adjusted until TIRF was achieved and the images were then collected through the Elements software (Nikon) using a Cascade 512B EM CCD camera (Photometrics).

Confocal images were collected on a Leica TCS SP5 AOBS inverted confocal using a 63x/0.60–1.40/HCX PL Apo objective. The confocal settings were as follows: pinhole 50 μm, scan speed 400 Hz unidirectional, frame average ×2. Images were collected sequentially using the following detection mirror settings: 405 nm (15%), 488 nm (50%) and 561 nm (30%) laser lines respectively. When acquiring 3D optical stacks the confocal software was used to determine the optimal number of Z sections.

## Author Contributions

N.T. and A.S. wrote the manuscript. A.S., F.C. and N.T. designed the study and provided supervision for R.R.K. and O.W.G.M. A.S. performed the epifluorescence microscopy experiments shown in figures 1,2,3, S2, S5, the ELISA (figure 1), TIRF (figure 4), and qPCR (figures 1 and S1) experiments, and the quantitative colocalisation analysis (figure S9). R.R.K. performed the epifluorescence microscopy experiments shown in figures 7, 8, 9, S6, S7, S8 and gel zymography (figure S4b). O.W.G.M. performed the immunofluorescence experiment shown in figure 6 and the gelatin degradation quantification study shown in figure S4a. R.D. acquired the AFM images (figure 4 and S3), A.T. acquired confocal microscopy images (figure 5).

## Supplementary Material

Supplementary Information Supplementary information
