# Supplementary material for: Fibronectin localization and fibrillization are affected by the presence of serum in culture media
Source: Sci Rep. 2015 Mar 23;5:9278. doi: 10.1038/srep09278 (PMC4369722; doi:10.1038/srep09278)
Supplement: Supplementary Information [file srep09278-s1.doc]

**Fibronectin localization and fibrillization are affected by the presence of serum in culture media**

Alessandro Siani^1^, Rong R. Khaw^2^, Oliver W.G. Manley^2^, Annalisa Tirella^1,2^, Francesco Cellesi^1^, Roberto Donno^2^, Nicola Tirelli^1,2^

^1^ Manchester Pharmacy School, University of Manchester, Oxford Road, Manchester, M13 9PT, United Kingdom

^2^ School of Medicine, Institute of Inflammation and Repair, University of Manchester, Oxford Road, Manchester, M13 9PT, United Kingdom

SUPPLEMENTARY INFORMATION

**Table S1^a^. Expression of most common MMPs in (myo)fibroblasts^b^. MMP-2 in bold.**

| Factor | MMPx | Effect | | Environment | Cells | References |
| --- | --- | --- | --- | --- | --- | --- |
| TGF-β1 | MMP-1 | **-** | ~5 fold | 1-10% FBS | HDF |  |
|  | MMP-1 | **+** | Non-quantified | 15% FBS | RA human synoviocytes | [^3^](#_ENREF_3) |
|  | MMP-1 | **-** | ~3 fold | SFM | Human keloid-derived fibroblasts | [^4^](#_ENREF_4) |
|  | **MMP-2** | **+** | ~2 fold |  |  |  |
|  | **MMP-2** | **-** | ~ 2.5 fold | SFM | Rat embryonic fibroblasts | [^5^](#_ENREF_5) |
|  | **MMP-2** | **-** | ~ 5 fold | SFM | Vascular SM cells ^c^ | [^6^](#_ENREF_6) |
|  | **MMP-2** | **+** | Non quantified | 10% FBS | A549 human alveolar cells ^d^ | [^7^](#_ENREF_7) |
|  | MMP-9 | **+** | Non quantified |  |  |  |
|  | **MMP-2** | **+** | ~3 fold | 10% FBS | Rat tubular epithelioid cells ^d^ | [^8^](#_ENREF_8) |
|  | MMP-14 ^e^ | **+** | ~3 fold |  |  |  |
|  | **MMP-2** | **+** | ~4 fold | *in vivo* | Mouse wound | [^9^](#_ENREF_9) |
|  | MMP-14 | **+** | ~3 fold |  |  |  |
|  | MMP-3 | **=**  **+** | No effect alone  ~3 fold with IL1α | SFM | Human cardiac fibroblasts | [^10^](#_ENREF_10) |
|  | MMP-3 | **-** | ~ 30% | 16% FBS | Rat pancreatic stellate cells | [^11^](#_ENREF_11) |
|  | MMP-9 | **-** | ~ 2 fold |  |  |  |
|  | MMP-2 | **=** | Non quantified | 0.3% FBS | HSC | [^12^](#_ENREF_12) |
|  | MMP-3 | **=** | Non quantified |  |  |  |
|  | MMP-14 ^e^ | **+** | Non quantified |  |  |  |
|  | MMP-14 ^e^ | **+** | ~1.7 fold | 10% FBS | HT-1080 human fibrosarcoma cells | [^13^](#_ENREF_13) |
| Tumor ^f^ | MMP-1 | **+** | ~2 fold | *in vivo* | Cancer-associated myofibroblasts | [^14^](#_ENREF_14) |
|  | MMP-9 | **+** | Not quantified | *in vivo* | PCa-associated myofibroblasts | [^15^](#_ENREF_15) |
| Mechanical tension ^f^ | MMP-1 | **+** | ~2.5 fold | SFM | Periodontal ligament fibroblasts | [^16^](#_ENREF_16) |
|  | **MMP-2** | **+** | ~10 fold | 10%FBS | HDF | [^17^](#_ENREF_17) |
| Keloid scars vs normal scars | **MMP-2** | **+** | ~2 fold | *In vivo* | Human keloid scars | [^18^](#_ENREF_18) |
|  |  | **+** | ~2.5 fold | 10% FBS | Human keloid- derived fibroblasts |  |

^a^ Abbreviations: FBS – Fetal Bovine Serum; HDF – Human Dermal Fibroblasts; Human Stellate Cells; PCa – Prostate Cancer; PDGF – Platelet-Derived Growth Factor; RA – Rheumatoid Arthritis; SFM – Serum-Free Medium; SM – Smooth Muscle; TGF-β1 - Transforming Growth Factor β1.

^b^ Analysis limited to MMP expression; a realistic picture should also include epigenetic, post-transcriptional and post-translational phenomena affecting MMP activity [^19^](#_ENREF_19), e.g. activation via cleavage of the ‘cysteine switch’ by furin, plasmin or other MMPs [^20-22^](#_ENREF_20), or the presence of inhibitors .

^c^ After pre-treatment with PDGF; a 2-fold decrease is recorded if PDGF is present in the medium.

^d^ From Epithelial-Mesenchymal Transition (EMT), therefore this datum may not be representative of fibroblast-myofibroblast differentiation.

^e^ Also known as MT-1 MMP (Membrane Type 1 Matrix MetalloProteinase).

^f^ Possibly due to TGF-β1 production in the tumour or its mechanically induced release from extracellular sites.

**1. Comparison of the effects of TGFβ1 exposure in murine cell lines and in HDFs**

**Methods.** L929 murine fibroblasts (ECACC) were purchased from Sigma-Aldrich (Gillingham, UK). NIH-3T3 murine embryonic fibroblasts (ATCC) were purchased from LGC standards (Teddington, UK). Cells were cultured using high glucose Dulbecco’s Modified Eagle Medium (DMEM, D6546, Sigma-Aldrich) supplemented with 10% v/v foetal bovine serum (FBS, Invitrogen) for L929, or 10% Newborn Calf Serum (NCS, Invitrogen) for NIH-3T3, 1% v/v penicillin/streptomycin solution (Sigma-Aldrich), 1% v/v L-glutamine (Invitrogen) and incubated under sterile conditions at 37°C/5% CO_2_. For all experiments, cells were seeded in the appropriate culture vessel and allowed to attach for 24 hours in presence of 10% serum. Subsequently, they were washed 3 times with warm serum-free medium (SFM), and treated for 48 hours with TGF-β1 (human recombinant, Abcam, UK).

HDFs were cultured as described in the main text.

**- RNA Extraction / qRT-PCR**. All reagents used in this section were purchased from Life Technologies (Carlsbad, California, USA) unless otherwise specified. Cells were seeded on TCPS in a 6 well plate at a density of 2x10^4^ cells/well and treated with TGF-β1 as described above, lysed with TRIzol® and the total RNA was extracted and purified by phenol-chloroform extraction followed by purification using the silica-cartridge system from PureLink™ RNA mini kit (Ambion®). Purified RNA was obtained via sequential elutions with RNase-free water and stored at -80°C for long term use. Total RNA concentration and purity were measured through spectrophotometry (NanoDrop® ND-1000; Thermo Fisher Scientific, Waltham, Massachusetts, USA). Samples with A260/280nm absorbance ratio in the range 1.80-2.0 were used for reverse transcription. The total RNA extracted as described above was reverse transcribed using High Capacitiy RNA-to-cDNA™ kit. Reverse transcription was performed using the Peltier Thermal Cycler PTC-200 (MJ Research, Waltham, Massachusetts, USA). The expression of myofibroblastic markers was quantified by real-time polymerase chain reaction. The following TaqMan assays were used for HDF: human α-SMA (Hs00426835_g1), human ED-A FN (Hs01549959_m1). Human GAPDH (Hs02758991_g1) was used as endogenous control. For NIH-3T3 and L929, SYBR green real time PCR was performed on murine cDNA using the following primers: α-SMA forward (CCACCGCAAATGCTTCTAAGT), α-SMA reverse (GGCAGGAATGATTTGGAAAGG), ED-A FN forward (ACAGGGTGACCTACTCGAGC), ED-A fibronectin reverse (GACTGTGTACTCAGACCCCG), GAPDH forward (GTTGTCTCCTGCGACTTCAAC), GAPDH reverse (TCATTGTCATACCAGGAAATGAGC). The qPCR reaction was performed on a StepOnePlusTM Real-Time PCR System (Applied Biosystems, Carlsbad, California, USA), and the relative expression of target genes was quantified using the StepOne software (Applied Biosystems). All qPCR experiments were performed in 3 different wells/RNA sample (technical replicates) and each experiment was repeated 3 times (biological replicates).

**Results**

We have compared the TGF-β1 response of Human Dermal Fibroblasts (HDFs) with that of two popular murine fibroblastic cell lines, NIH-3T3 and L929, cultured in 10% FBS (for HDF and L929) or 10% NCS (for NIH-3T3). The two cell lines presented peculiarities; for example, TGF-β1 is typically anti-mitotic for most cells[^25^](#_ENREF_25), but stimulated 3T3 proliferation (Figure 1SI), an effect also observed in other studies .

Further, the exposure of 3T3 and of L929 to up to 10 ng/mL TGF-β1 failed to induce a significant increase in the expression of typical myofibroblast markers such as Extra-Domain A fibronectin (ED-A FN; Figure S1B) or α-SMA (Figure S1C), although variable amounts of α-SMA-containing stress fibres can be recognised in most TGF-β1 treated 3T3 cells


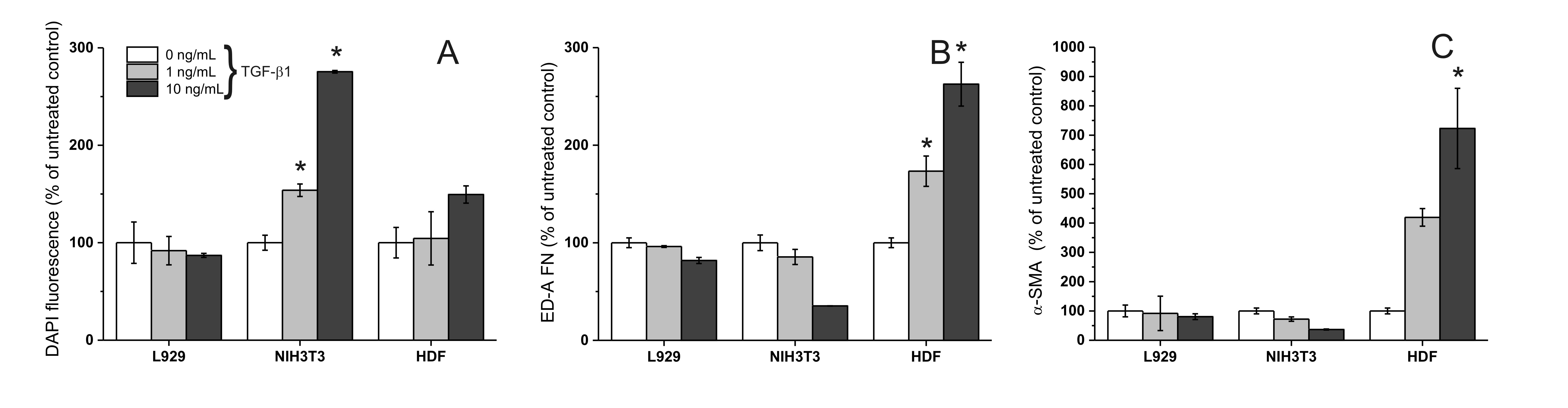


Figure S1 Effects of TGF-β1 treatment in murine cell lines vs. HDFs and in the presence of serum. *A:* DAPI emission can be assumed to be roughly proportional to the cell number; therefore, it provides a semi-quantitative indication of the differential proliferative activity seen in three fibroblast cell types after a 48 hours exposure to medium containing 10% serum and different concentrations of TGF-β1. The growth factor is typically non-mitogenic, therefore the proliferation observed on 3T3 cells (previously observed with Alamar Blue [^26^](#_ENREF_26) or Crystal Violet[^27^](#_ENREF_27) assays) is likely due to some form of secondary signalling. *B:* The expression of ED-A FN mRNA in the three cellular models (10% serum, 48 hours) shows a TGF-β1-induced upregulation of this myofibroblast marker only for HDFs. *C:* The expression of α-SMA mRNA in the three cellular models (10% serum, 48 hours) indicates that TGF-β1 induces an upregulation of also this myofibroblast marker only for HDFs Data are expressed as mean±S.D.; *n*=3.

**2. Influence of the serum starvation time on FN topology**

**
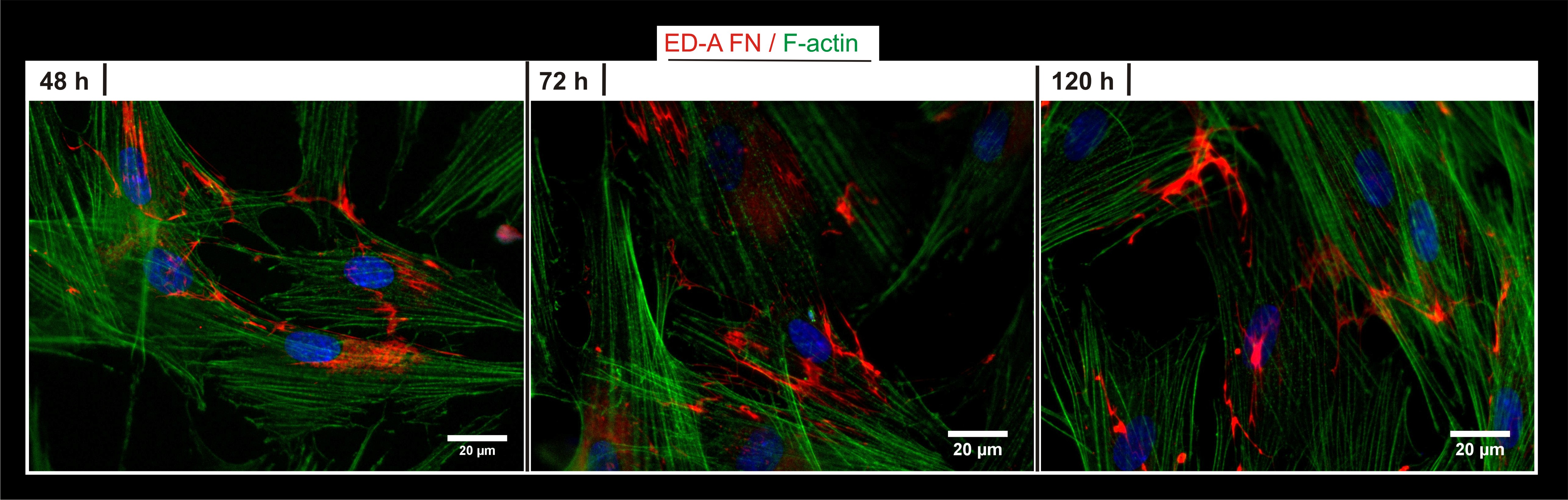
**

**Figure S2** Prolonging serum starvation from 2 to 5 days did not significantly alter the organization of cellular fibronectin (here stained in its ED-A variant using the IST-9 antibody). In order to minimize possible artefacts induced by prolonged starvation, in this study we have therefore used a starvation time of 2 days.

**3. AFM on cells**

**- Atomic Force Microscopy (AFM).** Human dermal fibroblasts were fixed in 4% paraformaldehyde for 15 minutes and then washed twice with PBS. Images of fixed cells were acquired in PBS at 25°C using a Molecular Force Probe 3D AFM (MFP-3D, Asylum Research, Santa Barbara, CA). AFM imaging was performed in contact mode with a scan rate of 0.5 Hz. A silicon nitride cantilever (model NP-10, cantilever D, Bruker, Camarillo, CA) with a nominal spring constant value of 0.06 N/m was employed. Images were analysed (e.g. profile extraction) using the Igor-pro software (Asylum Research AFM software, Version 101010+1202, Wavemetrics, Portland, OR).

**
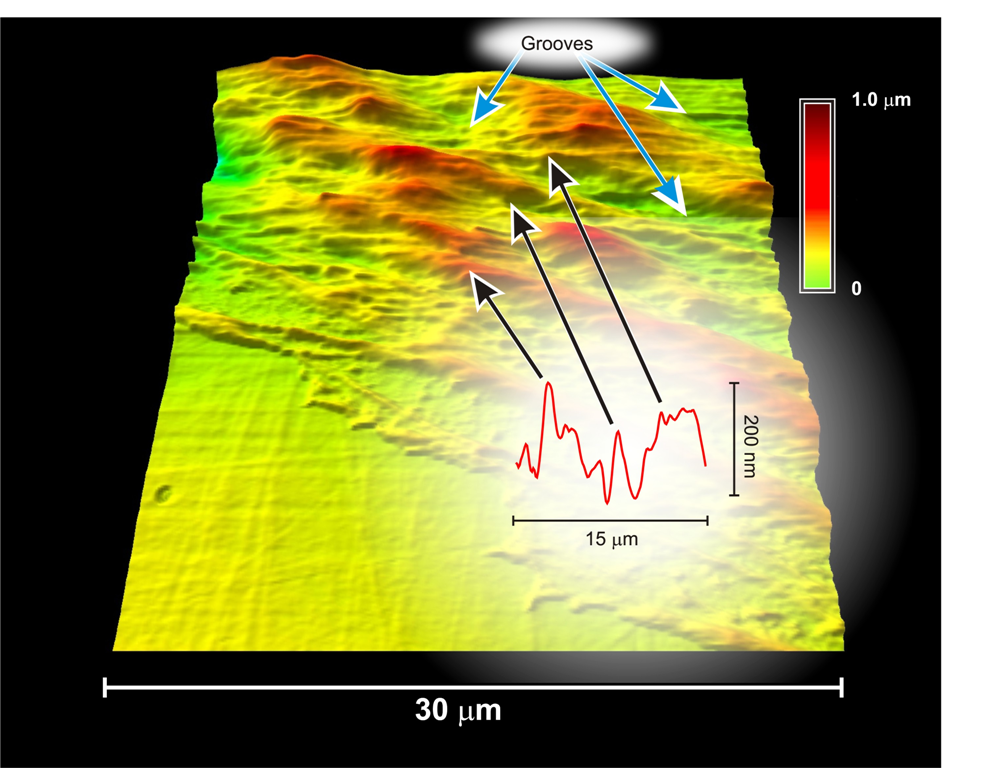
**

Figure S3 Contact mode AFM height image of part of the cell body of a HDF on a TCPS surface under serum-free conditions. The inset shows that the difference in height produced by the dorsal stress fibres are in the range of 100-200 nm.

**4. Gelatin degradation**

**Gel zymography.** Supernatants of HDF serum-free cultures treated with 10 ng/mL TGF-β1 (48 hours) as well as untreated controls were collected and snap-frozen using liquid nitrogen. Proteins were isolated and concentrated using the Amicon Ultra centrifugal filters (Millipore, Watford, UK). The total protein concentration was measured via the Bradford assay (Biorad, Hemel Hempstead, UK). Fresh serum-free DMEM containing 1% v/v penicillin/streptomycin solution (Sigma-Aldrich) and 1% v/v L-glutamine (Invitrogen) that had not been in contact with cell cultures, was used as the blanking buffer. Samples were prepared in a final volume of 40 μL containing an equal volume of protein solutions (1 mg/mL) and sample buffer (62.5 mM Tris-HCl, 25% v/v glycerol, 4% w/v sodium dodecyl sulphate (SDS) and 0.01% w/v Bromophenol Blue; pH 6.8). These samples were then loaded into a Criterion™ Precast Gel (10% Zymogram, gelatin; Bio-Rad, Berkeley, California). Precision Plus Protein™ Standards (Bio-Rad) was used as a molecular weight reference for the protein samples and to monitor protein migration during electrophoresis. Electrophoresis was performed at 125V constant, under non-reducing conditions using a Tris/Glycine/SDS running buffer (25 mM Tris-HCl, 192 mM glycine and 0.1% (w/v) SDS; pH 8.3) at room temperature. The gel was rinsed in deionised H2O under gentle agitation at room temperature for 30 minutes. The gel was incubated for 12 hours at 37°C in Zymogram Development Buffer containing 50 mM Tris-HCl, 200 mM NaCl, 5 mM CaCl2, 0.02% v/v Brij-35 at pH 7.5 (Bio-Rad) to activate enzymes after electrophoresis. The gel was subsequently stained with Coomassie Blue R-250, which contained 0.03% w/v Bromophenol Blue, 45% v/v methanol, 10% v/v acetic acid (Sigma-Aldrich) under gentle agitation for 1 hour at room temperature. Clear bands as a result of proteolytic activity were obtained by de-staining the gel with 45% (v/v) methanol, 10% (v/v) acetic acid for a further 2 hours at room temperature under gentle agitation. The gel was digitally imaged using the AlphaImagerTM 2200 (ProteinSimple, Santa Clara, California, USA) and protease activity was evaluated by densitometry analysis of the bands using Image J (National Institutes of Health, Bethesda, MD, USA). Zymography experiments were performed in triplicate using supernatants from 3 different wells (biological replicates).

**
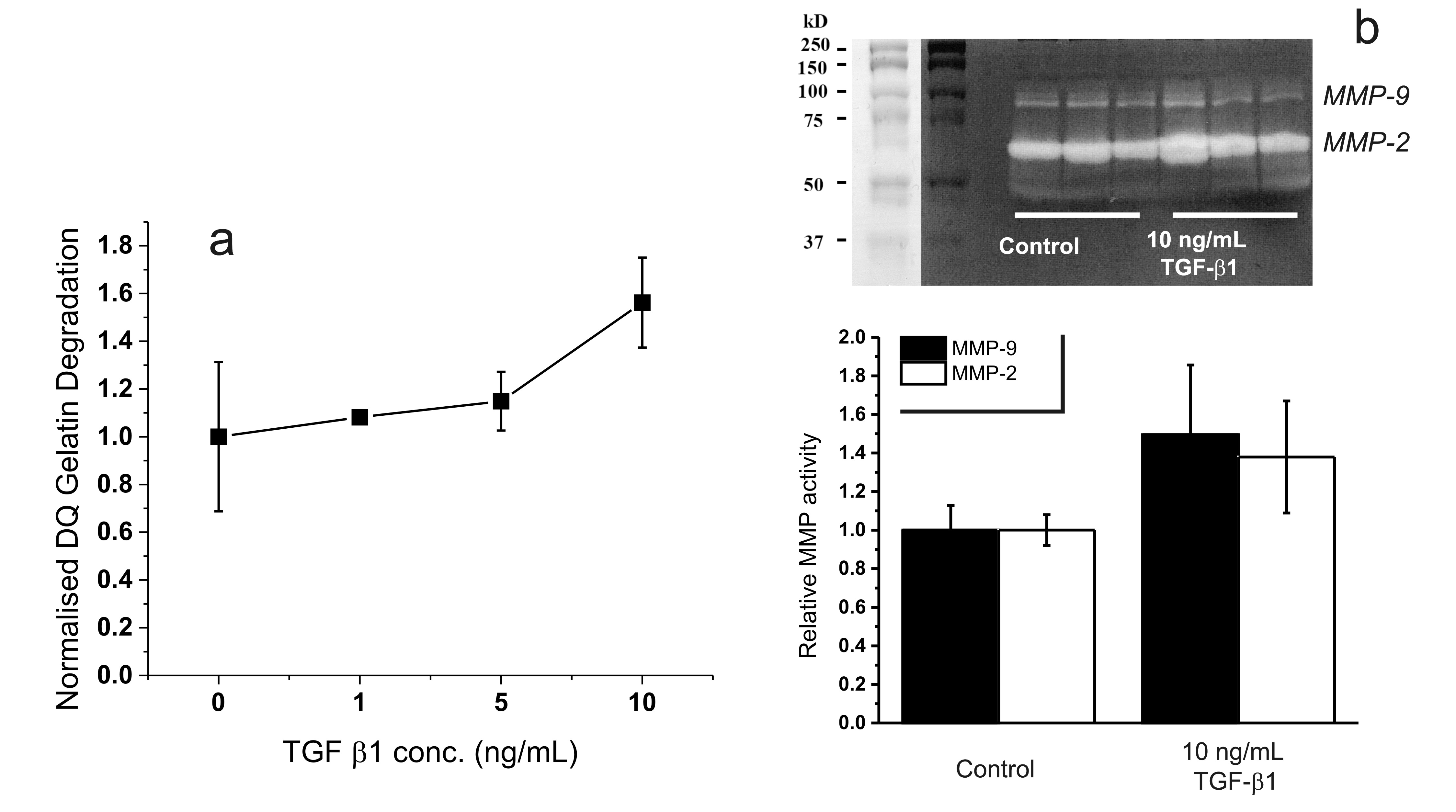
**

Figure S4 Gelatinolytic activity of HDFs. a*:* Effect of TGF-β1 concentration on the gelatin degradation activity (estimated from the fluorescence of DQ^TM^ gelatin) under serum-free conditions; please note that the normalization of gelatin degradation against the protein content of the sample provides an indication of the average gelatinolytic activity per cell. A qualitatively similar behavior can be seen in 10% FBS. *b:* *Top:* gelatinolytic proteins identified via SDS-PAGE zymography on a gelatin-containing gel; two bands can be observed for the ≈ 90 kDa component (MMP-9): the larger molecular weight bands are typically considered to be the zymogen forms of the two MMPs[^28^](#_ENREF_28). A similar structure is expected also for the ≈ 70 kDa component (MMP-2), but it is visible only on some of the samples; however, the poor separation between the two forms of MMP-2 is known[^29^](#_ENREF_29). *Bottom:* estimation of the relative MMP activity from the intensity of zymography bands. Although a certain TGF-β1-induced upregulation is visible for both MMP-2 and MMP-9 activity, Student’s t-test showed the difference not to be significant (P>0.05). Data are expressed as mean±S.D.; n=3*.*

### 5. Colocalization analysis

Colocalization data were generated using the Coloc2 plugin bundled with Fiji image analysis software (<http://fiji.sc/>). See section 2 in Supplementary Information / Additional Materials and Methods, for a more detailed explanation of how Pearson’s correlation coefficient and Mander’s colocalization split coefficients are calculated. In brief, Pearson’s correlation coefficient: +1 = perfect colocalization; 0 = no colocalization (i.e. random colocalization); -1 = perfect negative colocalization (i.e. perfect exclusion). For Mander’s colocalization split coefficients: 1 = perfect colocalization; 0 = no colocalization. Images were thresholded in order to remove background noise prior to generating the Mander’s colocalization coefficients for both channels (M_1_ and M_2_). Numerical data were presented as mean ± standard error of the mean (n=5).

### Calculation of colocalization coefficients

### A) Pearson’s Correlation Coefficient


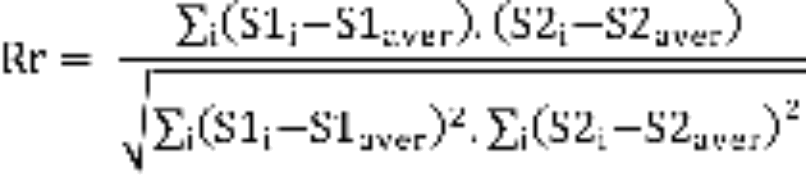


- **S1** = signal intensity of pixels in the first channel
- **S2** = signal intensity of pixels in the second channel
- **S1_aver_** = average pixel intensity of the first channel
- **S2_aver_** = average pixel intensity of the second channel
- For pixel i in the image of interest, S1 and S2 are intensities of the first and second channel, respectively.

Value of 1 = perfect colocalization; -1 = perfect exclusion; 0 = random colocalization.[^30^](#_ENREF_30)

### B) Mander’s Split Colocalisation Coefficients M_1_ and M_2_

The values obtained using Mander’s split colocalisation coefficients are normalized against total pixel intensity; this prevents problems often encountered whenabsolute pixel intensities are used. However, the Mander’s equation is sensitive to background noise and does not take into account the intensity of the second channel, other than assigning an intensity value of > 0. Hence, a threshold has to be set for both pixel intensities prior to performing the analysis ([http://www.macbiophotonics.ca](http://www.macbiophotonics.ca/)) [^31^](#_ENREF_31).


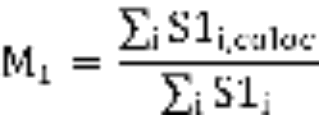


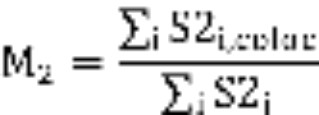


**S1_i,coloc_** = S1_i_ if S2_i_ > 0; S2 _i,coloc_ = S2_i_ if S2_i_ > 0

**S1_i, coloc_** = pixel intensity of first channel which is above the determined threshold.

**S2_i, coloc_** = pixel intensity of second channel which is above the determined threshold.

**Statistical analysis.** Data are expressed as mean±standard deviation. The two-samples Student’s t-test was used to test for statistical significance. P-values <0.05 were considered statistically significant.

**
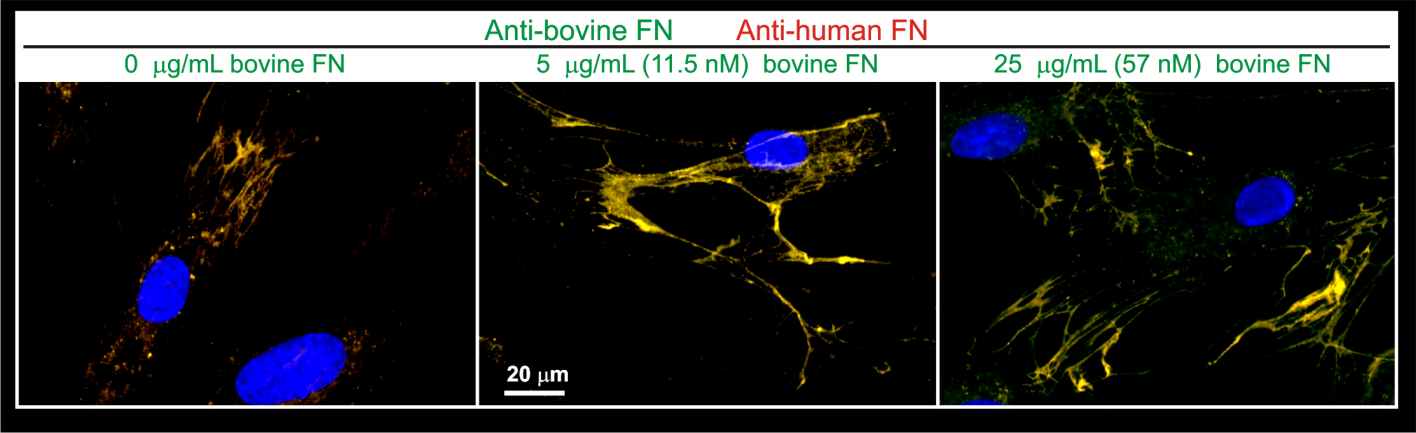
**

Figure S5 Immunostaining of HDFs treated with bovine FN. HDFs for 48 h were incubated with various concentrations of bovine FN in serum-free medium and stained with the monoclonal anti-human fibronectin FN15 and with the polyclonal anti-bovine fibronectin AB2047 antibodies. Note that the cross-species reactivity of AB2047 is clear from the green fluorescence of the control sample; this does not allow to ascertain the presence of bovine FN in the fibrillar meshwork.

**
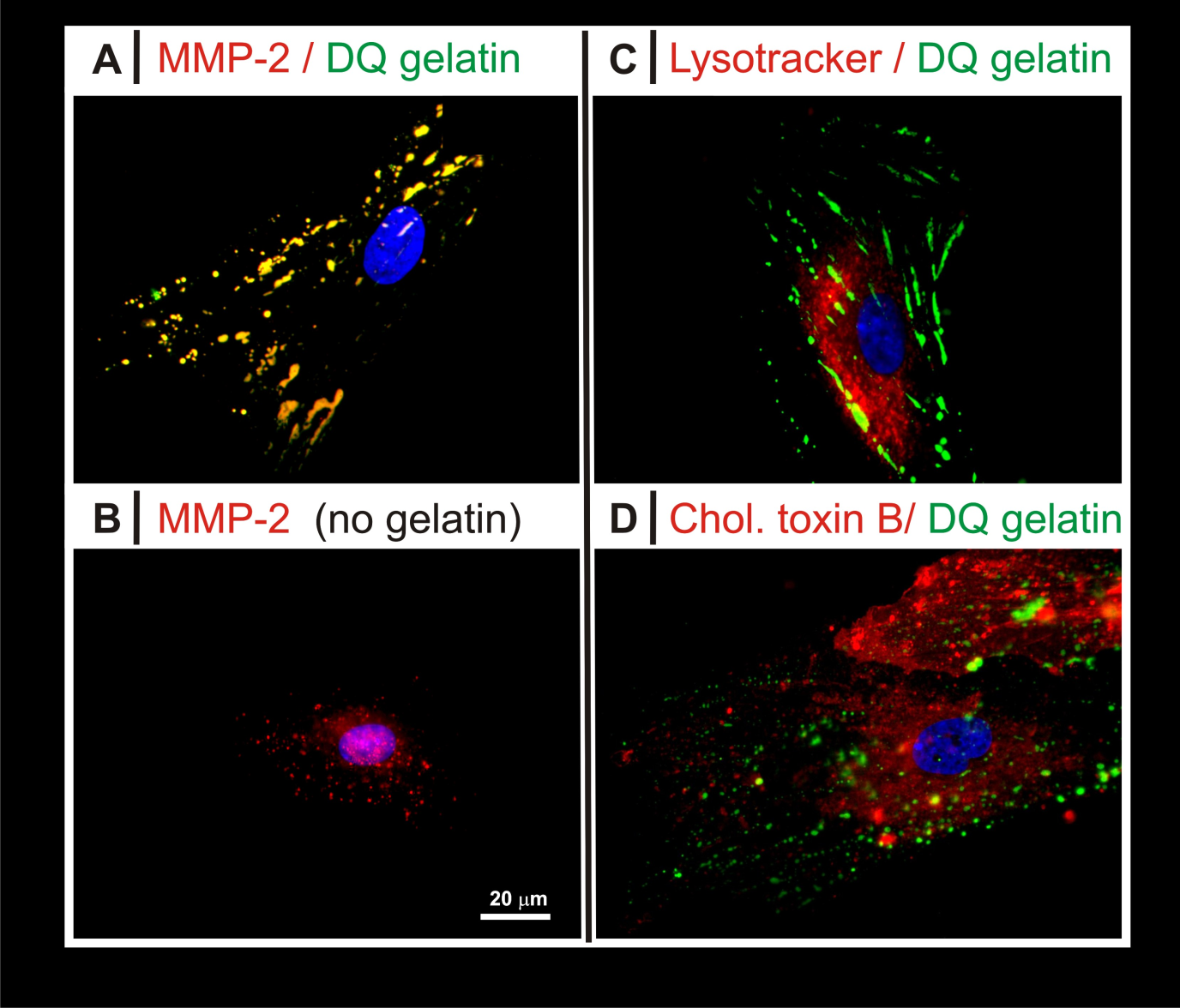
**

Figure S6 Fluorescence microscopy images of HDFs upon treatment with 10 ng/mL TGF-β1 and exposed to DQ^TM^ gelatin in 10% FBS. In all images green fluorescence corresponds to gelatin degradation. The red fluorescence corresponds to MMP-2 (rabbit polyclonal antibody and anti-rabbit Chromeo™) in *A* and *B*, to endolysosomal compartments (Lysotracker) in *C*, to lipid rafts (Cholera Toxin B) in *D*.

**
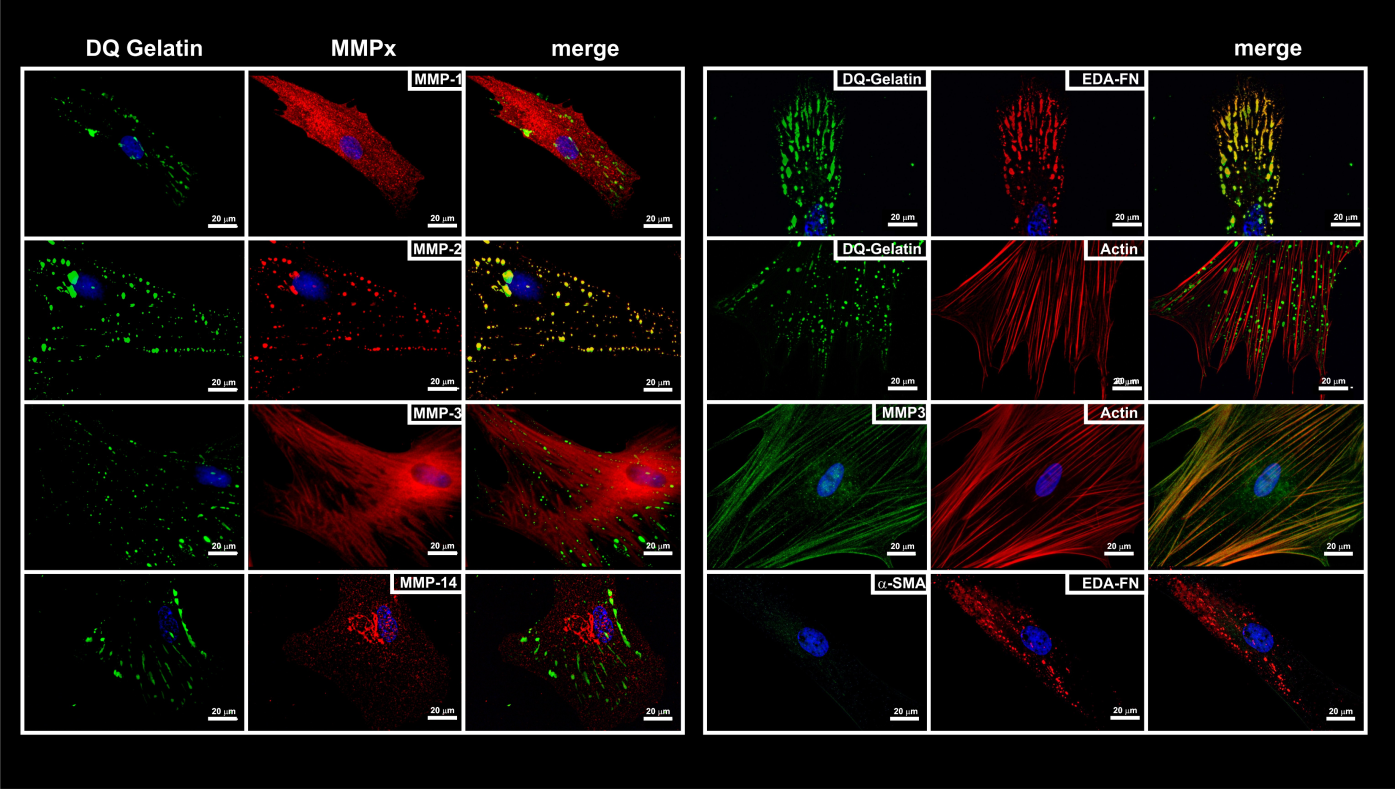
**

Figure S7 Colocalization fluorescence images for HDFs cultured in 10% FBS without TGF-β1. *Left panel*: DQ^TM^ gelatin showed almost perfect colocalization with MMP-2, an apparent mutual exclusion with MMP-3 and no specific relation to MMP-1 and MMP-14. *Right panel*: ED-A FN showed positive localization with DQ^TM^ gelatin (1^st^ row) and negative with F-actin (not with α-SMA because HDFs were not treated with TGF-β1). The latter, on the contrary, largely colocalized with MMP-3 (3^rd^ row).

**
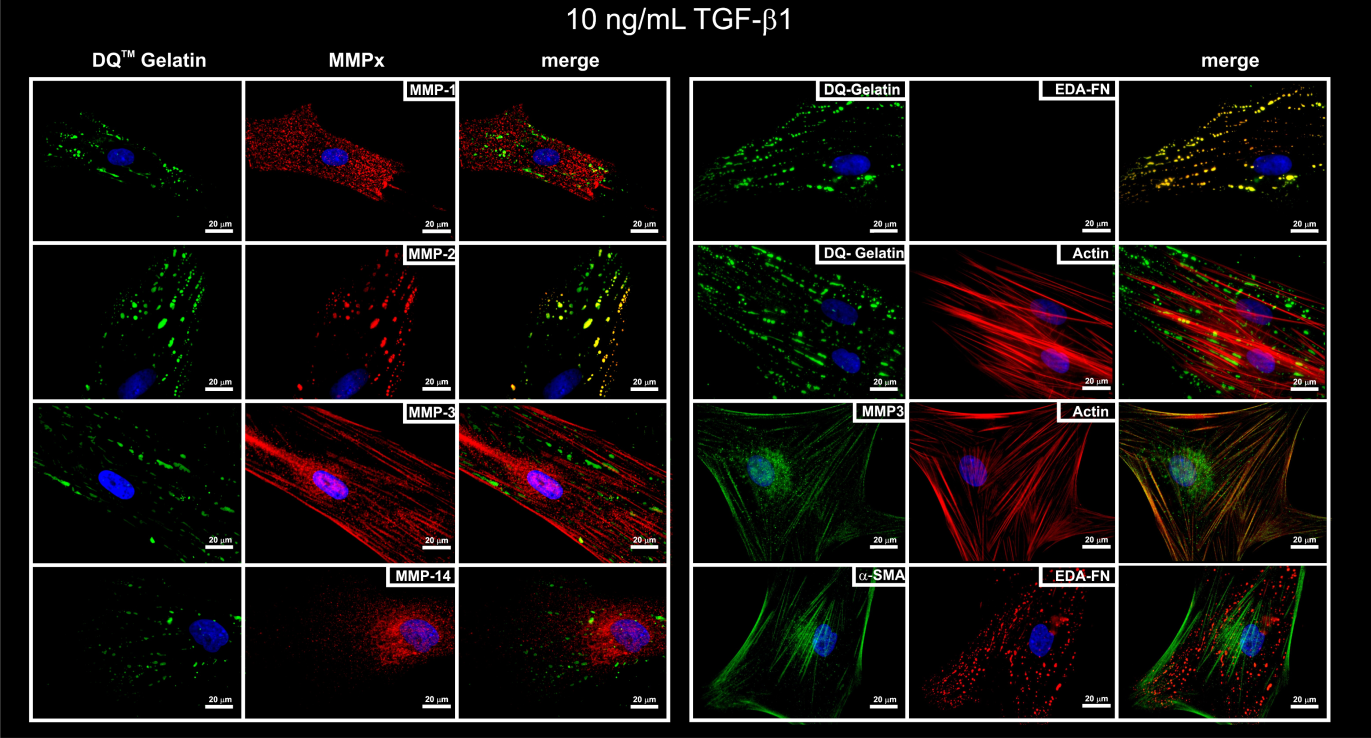
**

Figure S8 Colocalization fluorescence images for HDFs cultured in 10% FBS with 10 ng/mL TGF-β1. *Left panel*: DQ^TM^ gelatin showed almost perfect colocalization with MMP-2, an apparent mutual exclusion with MMP-3 and no specific relation to MMP-1 and MMP-14. *Right panel*: ED-A FN showed positive localization with DQ^TM^ gelatin and negative with α-SMA (4^th^ row) and F-actin (2^nd^ row). The latter, on the contrary, largely colocalized with MMP-3 (third row).

**Table S2.** Colocalization coefficients of MMPs and ED-A FN with DQ^TM^ gelatin and of MMP-3 and DQ^TM^ Gelatin with F-actin. Bold: positive colocalization; underlined and italics: negative colocalization. Data are expressed as mean±S.D.; n=5*.*

|  | Colocalization with DQ^TM^ Gelatin | | |
| --- | --- | --- | --- |
|  | Mander’s M1 | Mander’s M2 | Pearson’s |
| **EDA-FN** | **0.882±0.052** | **0.679±0.033** | **0.772±0.033** |
| MMP-1 | 0.374±0.030 | 0.183±0.046 | 0.169±0.011 |
| **MMP-2** | **0.962±0.008** | **0.667±0.079** | **0.847± 0.041** |
| *MMP-3* | *0.021±0.007* | *0.218±0.088* | *-0.876±0.027* |
| MMP-14 | 0.099±0.036 | 0.046±0.005 | 0.121±0.011 |
|  | Colocalization with F-actin | | |
|  | Mander’s M1 | Mander’s M2 | Pearson’s |
| **MMP-3** | **0.821±0.050** | **0.810±0.052** | **0.638±0.043** |
| *EDA-FN* | *0.246±0.060* (0.435±0.161^a^) | *0.162±0.053* (0.647±0.097^a^) | *-0.330±0.045*  (-0.100±0.187^a^) |
| *DQ^TM^ Gelatin* | *0.014±0.004* (*0.040±0.004^a^*) | *0.147±0.103* (*0.165±0.273^a^*) | *-0.836±0.119*  (*-0.868±0.212^a^*) |

^a^ in serum-free medium


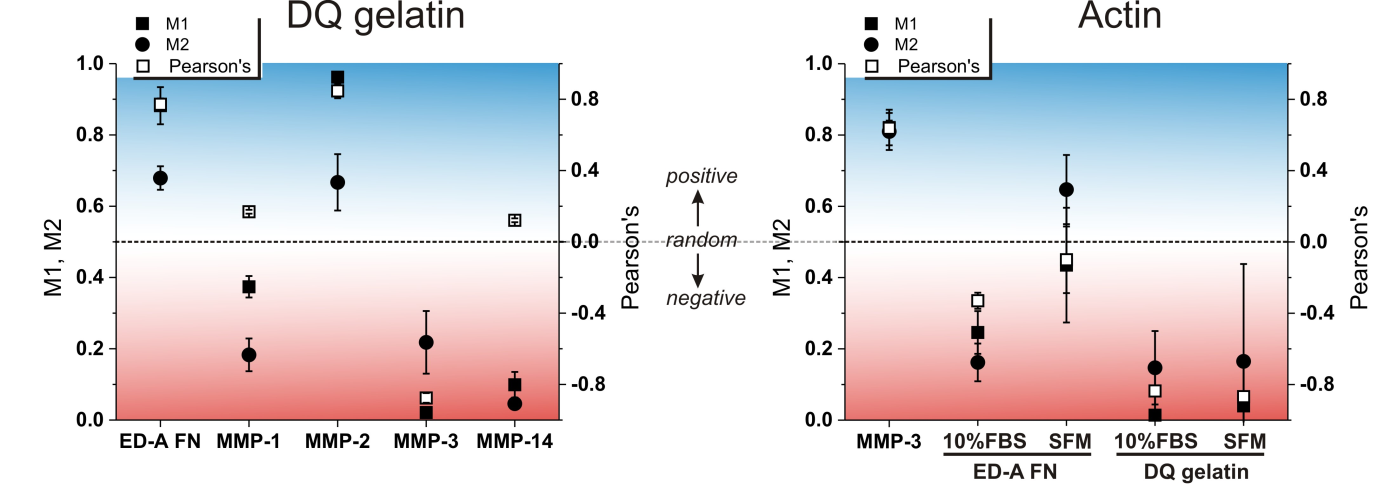


**Figure S9** Mander’s M1 and M3, and Pearson’s colocalization coefficients for various cellular components and DQ^TM^ gelatin **(*left*)** and actin **(*right*).** The vertical axes have been adjusted to report random colocalization in the middle of the scale; colours allow an easy identification of areas of strongly positive (blue) or negative (red) colocalization. Please note that the values for the MMP-14 - DQ^TM^ gelatin couple (left graph) may suggest a mutual exclusion, but we believe this to be an artefact due to the likely intracellular localization of MMP-14. For numerical values see Table S2. Data are expressed as mean±S.D.; n=5*.*

References

1 Igata, T., Jinnin, M. & Makino, T. Up-regulated type I collagen expression by the inhibition of Rac1 signaling pathway in human dermal fibroblasts. *Biochem. Biophys. Res. Commun.* 393, 101-105 (2010).

2 Yuan, W. & Varga, J. Transforming growth factor-beta repression of matrix metalloproteinase-1 in dermal fibroblasts involves Smad3. *J. Biol. Chem.* 276, 38502-38510 (2001).

3 Cheon, H. *et al.* Increased expression of pro-inflammatory cytokines and metalloproteinase-1 by TGF-beta 1 in synovial fibroblasts from rheumatoid arthritis and normal individuals. *Clin. Exp. Immunol.* 127, 547-552, doi:10.1046/j.1365-2249.2002.01785.x (2002).

4 Fujiwara, M., Muragaki, Y. & Ooshima, A. Keloid-derived fibroblasts show increased secretion of factors involved in collagen turnover and depend on matrix metalloproteinase for migration. *Br. J. Dermatol.* 153, 295-300 (2005).

5 Howard, E. W. *et al.* MMP-2 expression by fibroblasts is suppressed by the myofibroblast phenotype. *Exp. Cell Res.* 318, 1542-1553, doi:10.1016/j.yexcr.2012.03.007 (2012).

6 Risinger, G. M., Jr., Updike, D. L., Bullen, E. C., Tomasek, J. J. & Howard, E. W. TGF-beta suppresses the upregulation of MMP-2 by vascular smooth muscle cells in response to PDGF-BB. *Am. J. Physiol-Cell Ph.* 298, C191-C201, doi:10.1152/ajpcell.00417.2008 (2010).

7 Kasai, H., Allen, J. T., Mason, R. M., Kamimura, T. & Zhang, Z. TGF-beta 1 induces human alveolar epithelial to mesenchymal cell transition (EMT). *Resp. Res.* 6, doi:10.1186/1465-9921-6-56 (2005).

8 Cheng, S. F. & Lovett, D. H. Gelatinase A (MMP-2) is necessary and sufficient for renal tubular cell epithelial-mesenchymal transformation. *Am. J. Pathol.* 162, 1937-1949, doi:10.1016/s0002-9440(10)64327-1 (2003).

9 Cheon, S. S. *et al.* Beta-catenin regulates wound size and mediates the effect of TGF-beta in cutaneous healing. *FASEB J* 20, 692-701, doi:10.1096/fj.05-4759com (2006).

10 van Nieuwenhoven, F. A., Hemmings, K. E., Porter, K. E. & Turner, N. A. Combined effects of interleukin-1alpha and transforming growth factor-beta1 on modulation of human cardiac fibroblast function. *Matrix Biol.* 32, doi:10.1016/j.matbio.2013.03.008 (2013).

11 Shek, F. W. T. *et al.* Expression of transforming growth factor-beta 1 by pancreatic stellate cells and its implications for matrix secretion and turnover in chronic pancreatitis. *Am. J. Pathol.* 160, 1787-1798, doi:10.1016/s0002-9440(10)61125-x (2002).

12 Knittel, T. *et al.* Expression patterns of matrix metalloproteinases and their inhibitors in parenchymal and non-parenchymal cells of rat liver: regulation by TNF-alpha and TGF-beta 1. *J. Hepatol.* 30, 48-60, doi:10.1016/s0168-8278(99)80007-5 (1999).

13 Lohi, J., Lehti, K., Westermarck, J., Kahari, V. M. & KeskiOja, J. Regulation of membrane-type matrix metalloproteinase-1 expression by growth factors and phorbol 12-myristate 13-acetate. *Eur. J. Biochem.* 239, 239-247, doi:10.1111/j.1432-1033.1996.0239u.x (1996).

14 Holmberg, C. *et al.* Mapping proteolytic processing in the secretome of gastric cancer-associated myofibroblasts reveals activation of MMP-1, MMP-2, and MMP-3. *J Proteome Res* 12, 3413-3422, doi:10.1021/pr400270q (2013).

15 Gravina, G. L. *et al.* Phenotypic characterization of human prostatic stromal cells in primary cultures derived from human tissue samples. *Int. J. Oncol.* 42, 2116-2122, doi:10.3892/ijo.2013.1892 (2013).

16 Kook, S. H., Jang, Y. S. & Lee, J. C. Involvement of JNK-AP-1 and ERK-NF-κB signaling in tension-stimulated expression of Type I collagen and MMP-1 in human periodontal ligament fibroblasts. *J. Appl. Physiol.* 111, 1575-1583 (2011).

17 Karamichos, D., Brown, R. A. & Mudera, V. Collagen stiffness regulates cellular contraction and matrix remodeling gene expression. *J. Biomed. Mater. Res. A* 83, 887-894 (2007).

18 Imaizumi, R. *et al.* Promoted activation of matrix metalloproteinase (MMP)-2 in keloid fibroblasts and increased expression of MMP-2 in collagen bundle regions: implications for mechanisms of keloid progression. *Histopathology* 54, 722-730 (2009).

19 Chernov, A. V., Sounni, N. E., Remacle, A. G. & Strongin, A. Y. Epigenetic control of the invasion-promoting MT1-MMP/MMP-2/TIMP-2 axis in cancer cells. *J. Biol. Chem.* 284, 12727-12734 (2009).

20 Murphy, G. & Knauper, V. Relating matrix metalloproteinase structure to function: why the "hemopexin" domain? *Matrix Biol* 15, 511-518 (1997).

21 Cao, J., Rehemtulla, A., Bahou, W. & Zucker, S. Membrane type matrix metalloproteinase 1 activates pro-gelatinase A without furin cleavage of the N-terminal domain. *J. Biol. Chem.* 271, 30174-30180 (1996).

22 Pei, D. & Weiss, S. J. Furin-dependent intracellular activation of the human stromelysin-3 zymogen. *Nature* 375, 244-247 (1995).

23 Gomez, D. E., Alonso, D. F., Yoshiji, H. & Thorgeirsson, U. P. Tissue inhibitors of metalloproteinases: structure, regulation and biological functions. *Eur J Cell Biol* 74, 111-122 (1997).

24 Nagase, H., Visse, R. & Murphy, G. Structure and function of matrix metalloproteinases and TIMPs. *Cardiovasc. Res.* 69, 562-573 (2006).

25 Ten Dijke, P. T., Goumans, M. J., Itoh, F. & Itoh, S. Regulation of cell proliferation by Smad proteins. *J. Cellul. Physiol.* 191, 1-16, doi:10.1002/jcp.10066 (2002).

26 Voytik-Harbin, S. L., Brightman, A. O., Kraine, M. R., Waisner, B. & Badylak, S. F. Identification of extractable growth factors from small intestinal submucosa. *J. Cell. Biochem.* 67, 478-491, doi:10.1002/(sici)1097-4644(19971215)67:4<478::aid-jcb6>3.0.co;2-p (1997).

27 Ramirez, A. M. *et al.* Vitamin D inhibition of pro-fibrotic effects of transforming growth factor beta 1 in lung fibroblasts and epithelial cells. *J Steroid Biochem Mol Biol* 118, 142-150, doi:10.1016/j.jsbmb.2009.11.004 (2010).

28 Galis, Z. S., Sukhova, G. K., Lark, M. W. & Libby, P. Increased expression of matrix metalloproteinases and matrix-degrading activity in vulnerable regions of human atherosclerotic plaques *J. Clin. Invest.* 94, 2493-2503, doi:10.1172/jci117619 (1994).

29 Frederiks, W. M. & Mook, O. R. F. Metabolic mapping of proteinase activity with emphasis on in situ zymography of gelatinases: Review and protocols. *J. Histochem. Cytochem.* 52, 711-722, doi:10.1369/jhc.4R6251.2004 (2004).

30 Smallcombe A. Multicolor imaging: the important question of co-localization. *Biotechniques* 30, 1240-1242 (2001).

31 Manders, E. M. M., Verbeek, F. J. & Aten, J. A. Measurement of co-localisation of objects in dual-colour confocal images. *J. Microsc.* 169, 375-382 (1993).
